# Seasonally varying footprint of climate change on precipitation in the Middle East

**DOI:** 10.1038/s41598-018-22795-8

**Published:** 2018-03-13

**Authors:** Hossein Tabari, Patrick Willems

**Affiliations:** 1 Hydraulics Division, Department of Civil Engineering, KU Leuven, Kasteelpark Arenberg 40, BE-3001 Leuven, Belgium; 2 Department of Hydrology and Hydraulic Engineering, Vrije Universiteit, Brussel, Belgium

## Abstract

Climate change is expected to alter precipitation patterns; however, the amplitude of the change may broadly differ across seasons. Combining different seasons may mask contrasting climate change signals in individual seasons, leading to weakened signals and misleading impact results. A realistic assessment of future climate change is of great importance for arid regions, which are more vulnerable to any change in extreme events as their infrastructure is less experienced or not well adapted for extreme conditions. Our results show that climate change signals and associated uncertainties over the Middle East region remarkably vary with seasons. The region is identified as a climate change hotspot where rare extreme precipitation events are expected to intensify for all seasons, with a “highest increase in autumn, lowest increase in spring” pattern which switches to the “increase in autumn, decrease in spring” pattern for less extreme precipitation. This pattern is also held for mean precipitation, violating the “wet gets wetter, dry gets drier” paradigm.

## Introduction

There is now an overwhelming consensus among climate scientists that the frequency and intensity of extreme events will increase under the future climatic change^1–4^. Yet, there has not been a coherent picture for seasonal distribution of this increasing signal neither worldwide nor on a regional scale. It is specifically unclear whether extreme precipitation will be intensified for all seasons. Combining climate change impact of all seasons may lead to compensation of increasing and decreasing signals in the individual seasons and understate the expected changes and related damages to society in terms of human health and mortality and to the ecosystem.

Another important issue in this context is that climate change results are highly model dependent so that even opposite climate change signals may be achieved from different types of climate models^5–7^. This is mainly due to the fact that climate models with different resolutions and physics provide different representations of surface heterogeneities and mesoscale climatological structures^8–10^. To better understand the added value of fine-scale climate models versus coarse-scale ones, it is important to quantify the extent to which the discrepancy between driving and driven climate models varies with seasons.

In contrast to the projected increase in extreme precipitation, there is no robust change in precipitation totals on the global scale^11,12]^. Nevertheless, at the seasonal scale, “wet season becomes wetter, dry season becomes drier” paradigm for precipitation totals has been found in the literature^13–15^, following a similar physical reasoning explaining the “wet regions get wetter, dry regions get drier”^16–19^ or the rich-get-richer mechanism^20^. Yet, some researchers believe that “dry gets drier, wet gets wetter” paradigm has been overestimated and a large part of the land area experience an opposite paradigm^21–23^. Such contradicting results are likely connected to the type of data/model used^23^ and the transition of humid regions towards drier conditions or vice versa under future climate conditions^19,23^. Disputable is which paradigm is valid for future climate change projections derived from regional climate models (RCMs) with an improved representation of small-scale processes and local features.

Here we analyze how climate change signals and the associated uncertainties as well as the difference between the results of RCMs and driving global climate models (GCMs) for extreme and mean precipitation vary with seasons. An ensemble of WAS-CORDEX RCM projections (see Supplementary Table S1) is used to derive the change signals for the late 21st century. The domain of analysis is the Middle East region which has received less attention globally and regionally despite its high sensitivity to climate change due to an arid climate and limited resources to acclimate with the negative effects socially, economically and technologically.

## Results and Discussion

### How do climate change signals vary with seasons?

**Figure 1:**
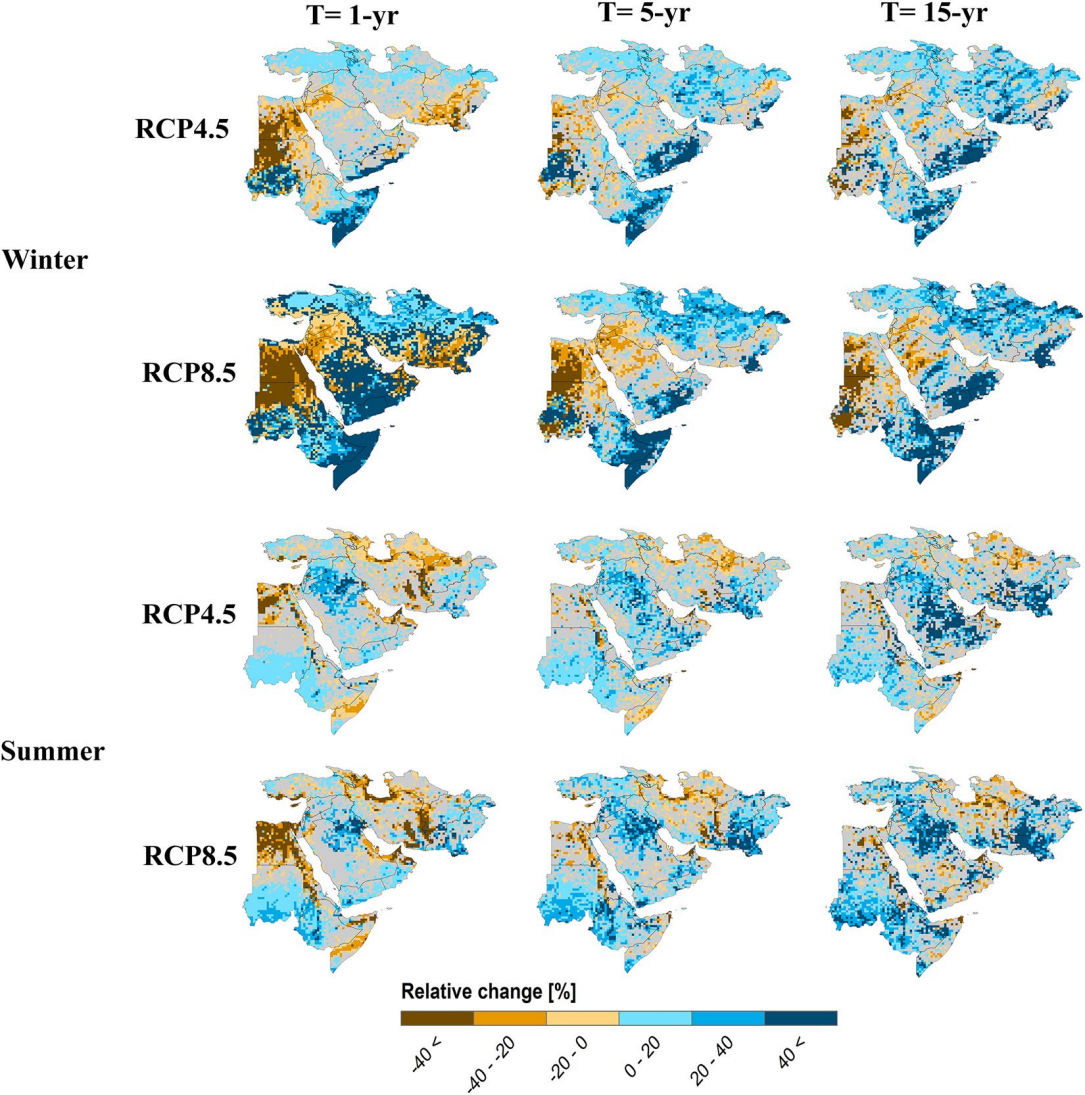
Changes in extreme precipitation of 15-, 5- and 1-year return periods (T) in extreme seasons (i.e., winter and summer) based on the median ensemble of the WAS-CORDEX RCMs for RCP4.5 and RCP8.5 over the Middle East region. The changes are computed for the period 2070–2099 with respect to the reference 1971–2000. Values are masked in gray where the change is not robust (change is robust if at least 70% of all model runs agree on the sign of the change). The maps were generated using the software ArcGIS (version 10) http://www.esri.com/products.

**Figure 2:**
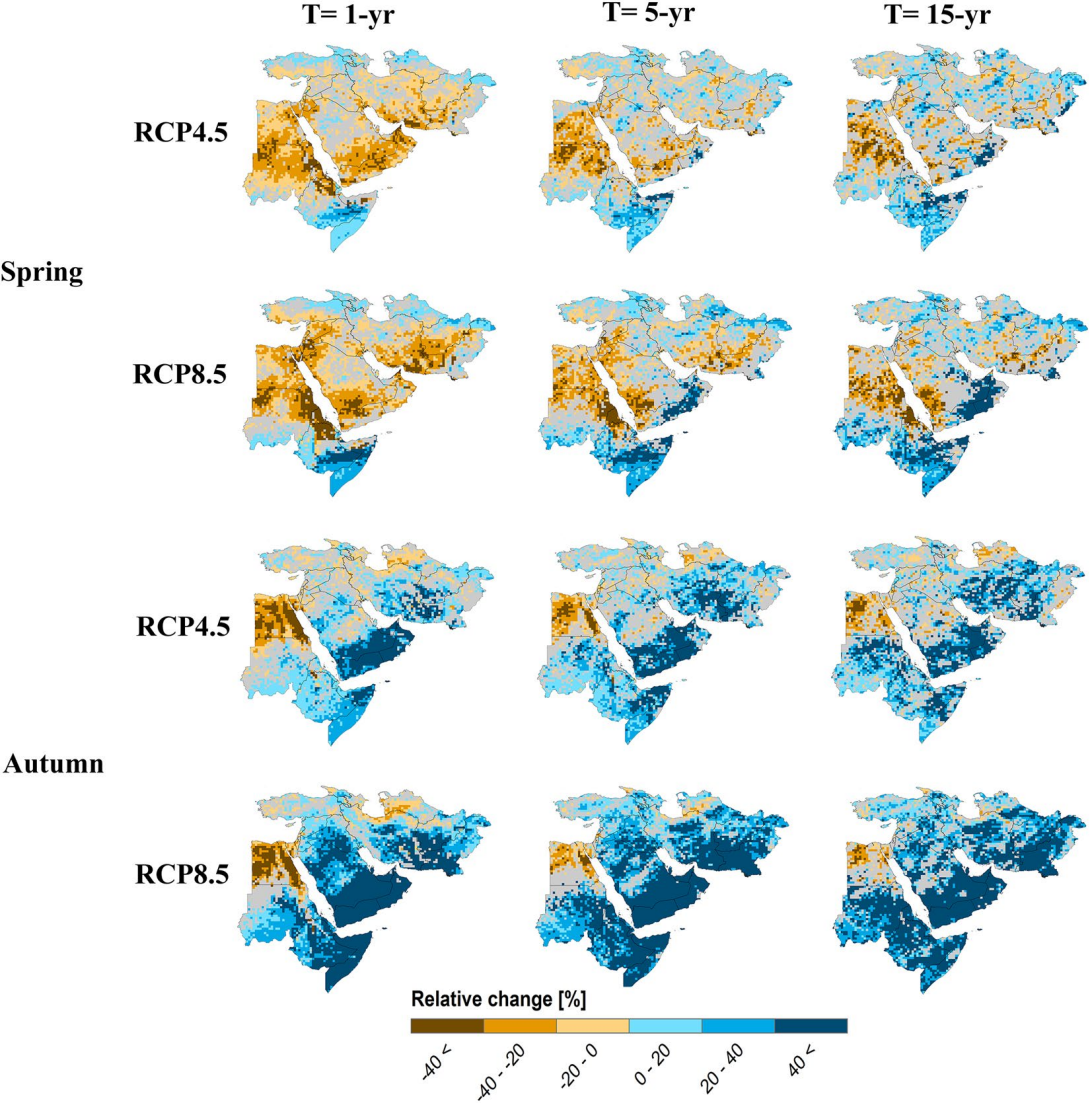
Changes in extreme precipitation of 15-, 5- and 1-year return periods (T) in transitional seasons (i.e., spring and autumn) based on the median ensemble of the WAS-CORDEX RCMs for RCP4.5 and RCP8.5 over the Middle East region. The changes are computed for the period 2070–2099 with respect to the reference 1971–2000. Values are masked in gray where the change is not robust (change is robust if at least 70% of all model runs agree on the sign of the change). The maps were generated using the software ArcGIS (version 10) http://www.esri.com/products.

Figures 1 and 2 show changes in extreme precipitation in different seasons based on the median ensemble of the WAS-CORDEX RCMs for RCP4.5 and RCP8.5 over the Middle East region. For the rare extreme events (extreme precipitation of 15-year return period), an increase is projected for all seasons, with a noticeably higher increase for autumn. The robust increase in the autumn rare events for RCP8.5 is observed in about 74% of the domain (Figure S2), while robust increasing signals cover a smaller part of the domain in spring (33% for RCP8.5). This “highest increase in autumn, lowest increase in spring” pattern gradually changes to “increase in autumn, decrease in spring” pattern for less extreme precipitation (i.e., very extreme and moderate extreme). The decreasing signal in spring very extreme and moderate extreme precipitation is not as strong as the increasing signal for autumn in terms of magnitude and spatial extent. Consistent over different seasons and precipitation intensities, the amplitude of the changes is stronger in the most pessimistic emission scenario RCP8.5 than in the intermediate scenario RCP4.5 (Figure S2).

**Figure 3:**
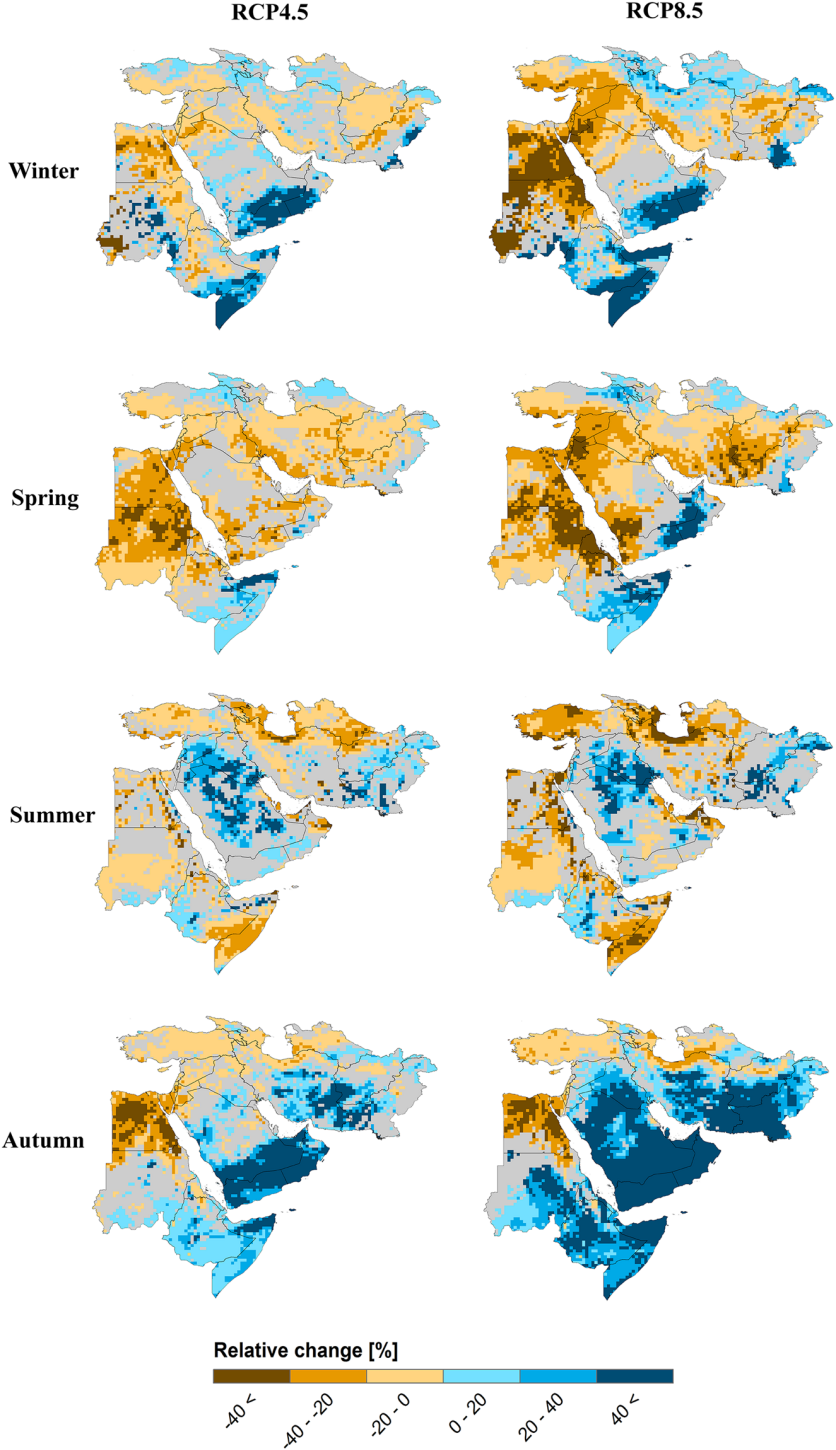
Changes in mean precipitation in different seasons based on the median ensemble of the WAS-CORDEX RCMs for RCP4.5 and RCP8.5 over the Middle East region. The changes are computed for the period 2070–2099 with respect to the reference 1971–2000. Values are masked in gray where the change is not robust (change is robust if at least 70% of all model runs agree on the sign of the change). The maps were generated using the software ArcGIS (version 10) http://www.esri.com/products.

The “increase in autumn, decrease in spring” pattern for less extreme precipitation is held for mean precipitation (Fig. 3). For RCP8.5, over 66% of the land area is affected by a robust increase in autumn mean precipitation, whereas around 58% of the land area shows a robust decrease in spring mean precipitation. The decrease in large-scale precipitation in the Middle East can be due to polewards movement of the Northern Hemisphere storm tracks under climate change conditions which weakens the Mediterranean storm track and reduces the number of cyclones crossing the Mediterranean^24–26^. There is a less spatially homogeneous change over the Middle East region for winter and summer mean precipitation and both increasing and decreasing signals are observed. Overall, the results of this study are in contrast to the “wet season gets wetter, dry season gets drier” paradigm and instead suggest “wetter autumn, drier spring” pattern. It means that the rainy season in the Middle East is shifting as a consequence of climate change. In fact, the rains will come too late and out of the growing season of crops. The decrease in spring precipitation obtained here along with the highly evaporative conditions in this season^27,28^ provide unfavorable conditions for rain-fed agriculture particularly of cereals, as spring crops rely on soil moisture derived from springtime rains or snowmelt^29,30^. On the other hand, the rainfall at an unexpected time together with more intense rainfall in the harvesting season will harm crop yield and degrade food security in the region. The Red Sea region (e.g., Egypt and Sudan) are seen as hotspots where a robust and large spring precipitation decrease is pronounced, while the hotspots for summer precipitation are the northern regions of the Middle East (e.g., Turkey and North Iran). The shift in rainy season (and available water) from winter and spring to autumn may exacerbate existing water management issues (e.g., water stress and dry zone expansion) across the Middle East. In addition to seasonal variation of climate change signals, the robust changes also accelerate with the emission level (from RCP4.5 to RCP8.5): from 57% to 69% of the land area for winter, from 68% to 74% for spring, from 54% to 57% for summer and from 67% to 74% for autumn (Figure S2).

The seasonal varying impact of climate change on mean and extreme precipitation over the Middle East region may be hidden by combining all seasonal values as pointed out in IPCC AR5^2^. At the annual scale, a decrease in annual precipitation totals has been found using the simulations of the CMIP3 GCMs under the A2 emissions scenario for the entire Middle East region except for the southern part with a slight increasing signal^31^.

### How do uncertainties in climate change signals vary with seasons?

**Figure 4:**
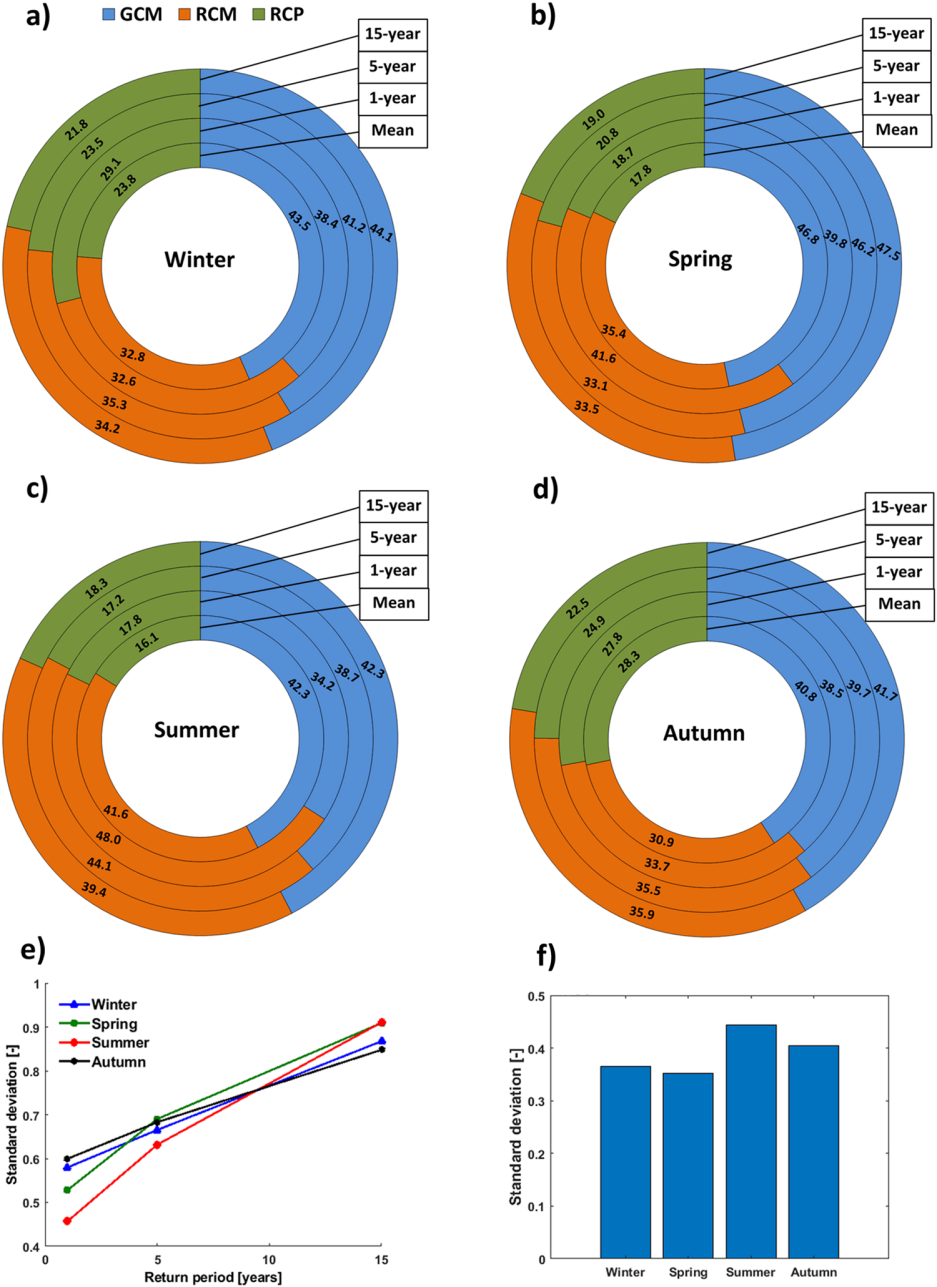
Donut charts of the fraction of total uncertainty in extreme (**e**) and mean (**f**) precipitation changes explained by GCM (blue), RCM (brown) and RCP (green) uncertainties for (**a**) winter, (**b**) spring, (**c**) summer and (**d**) autumn over the Middle East region. T = 15 yr, T = 5 yr and T = 1 yr refer to extreme precipitation of 15-, 5- and 1-year return periods, respectively. ‘Mean’ corresponds to mean precipitation.

Figures 4e,f show that the magnitude of uncertainty in precipitation changes widely differs across seasons. For both mean and extreme precipitation, the smallest amount of total uncertainty is seen for spring and winter, respectively. The largest uncertainty in rare extreme event changes is observed for summer and spring, and the total uncertainty decreases towards less extreme precipitation (Fig. 4e) and mean precipitation (Fig. 4f).

We decompose the total uncertainty in mean and extreme precipitation changes into GCM, RCM and RCP uncertainties (Fig. 4). The choice of GCMs is the dominant source of uncertainty in mean and extreme precipitation changes for all seasons except for summer for which RCM uncertainty dominates the total uncertainty for very extreme and moderate extreme precipitation. The dominancy of RCM uncertainty for summer extreme precipitation is in good agreement with the results found in Europe and North America^32–35^. This is attributed to the fact that local processes are mainly responsible for summer extreme precipitation changes rather than large-scale atmospheric circulations and each RCM has a different representation of such processes^35–38^. The RCP uncertainty is generally less prominent compared with the other uncertainty sources, being larger in the cold season (autumn and winter) than in the warm season (spring and summer). The RCP uncertainty accounts for 16–29% of the total uncertainty, whereas model uncertainties go as high as 48%. This is also evident from the spatial maps of relative importance of these three uncertainty sources (Figures S3-S6). The small scenario uncertainty in the CORDEX ensembles is due in part to the unavailability of the RCM simulations for the extreme low emission scenario (i.e., RCP2.6), which results in an underestimation of the scenario uncertainty^39,40^.

### How do downscaling signals vary with seasons?

**Figure 5:**
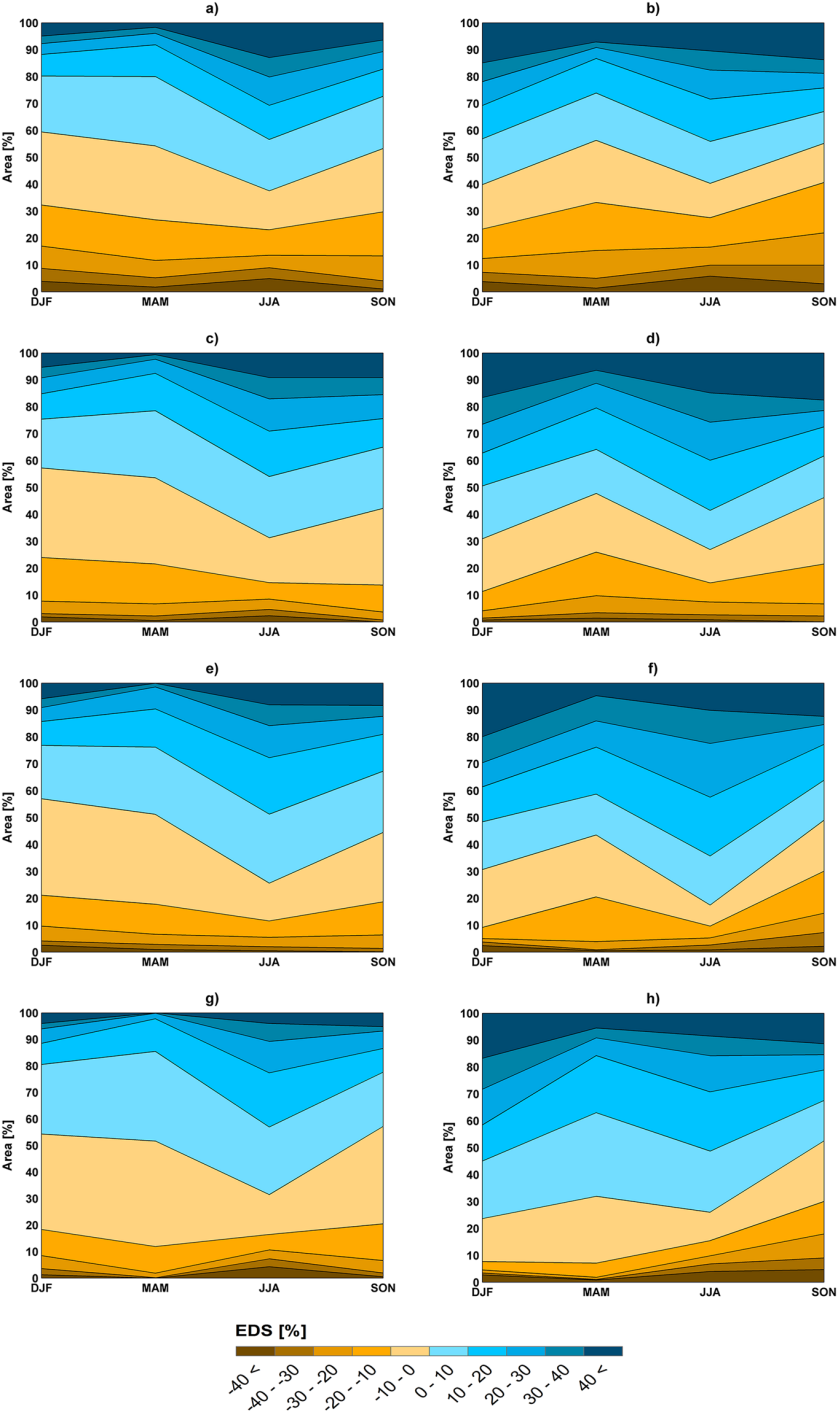
Percentage of the Middle East area with different classes of the ensemble downscaling signal (EDS) for mean (**g**,**h**) and extreme precipitation of (**a**,**b**) 15-, (**c**,**d**) 5- and (**e**,**f**) 1-year return periods for RCP4.5 (**a**,**c**,**e**,**g**) and RCP8.5 (**b**,**d**,**f**,**h**).

To compare the climate change signals from the WAS-CORDEX RCMs and the driving CMIP5 GCMs, the ensemble downscaling signal (EDS) is calculated, with a positive (negative) EDS denoting a larger (smaller) change by RCMs compared to driving GCMs (see Methods for the computational procedure). The results show seasonal differences in EDS of mean and extreme precipitation (Fig. 5). There are also large spatial variations in EDS over the Middle East region (Figures S7–S10). The largest portion of the study region with a positive EDS of mean and extreme precipitation is found for summer, implying greater changes by the WAS-CORDEX RCMs compared to the driving CMIP5 GCMs. Larger changes of summer precipitation from finer scale climate models have also been reported in other parts of the worlds^35,41,42^. The positive EDS for summer covers between 60% and 83% of the land area. Moreover, there is the lowest consistency (land area with −10≤ EDS ≤10) between the results of the RCM and the GCMs for both mean and extreme precipitation in summer (Figure S11). The discrepancy between the RCM and GCM results is because of their different parameterizations of local topographical forcings and different responses to sea surface temperature changes, the land–atmosphere interaction and the soil moisture/precipitation feedback^6,43^. Winter has the smallest area fraction with a positive EDS for RCP4.5, indicating smaller changes from the WAS-CORDEX RCMs for this emission scenario in comparison to the driving CMIP5 GCMs. For RCP8.5, the smallest land fraction with a positive EDS is found in spring for rare extreme and very extreme events and in autumn for moderate extreme and mean precipitation. In terms of consistency (land area with −10≤ EDS ≤10), there is the highest consistency between the GCM and RCM results for spring (Figures S11 and S12). This shows the low sensitivity of the climate change signals of extreme and mean precipitation to the physics and the internal dynamics of the models^44^ in spring, while the signals are highly sensitive in summer. Moreover, the consistency generally decreases from mean precipitation to rare extreme events for all seasons and from RCP4.5 to RCP8.5 for all seasons except for Autumn.

### Key findings and implications

This study provides an evidence for violation of “wet gets wetter, dry gets drier” paradigm using an ensemble of WAS-CORDEX RCM projections. Our results show an increase in autumn mean precipitation and a decrease in spring mean precipitation in the Middle East for the end of this century, while there is not a spatially coherent pattern for summer and winter precipitation changes. It means neither the wet season gets wetter nor the dry season gets drier in the region. As for extreme precipitation, the Middle East region is identified as a climate change hotspot where extreme precipitation is expected to increase for all seasons, with a “highest increase in autumn, lowest increase in spring” pattern. Extreme precipitation change signals are amplified for more extreme precipitation and higher emission level (RCP8.5). These results are alarming in the sense that arid regions such as the Middle East are more vulnerable to the future increase in extreme precipitation as their infrastructure is less experienced or not well adapted for extreme events. A well-adapted system on which people’s livelihoods can be based is needed for more erratic precipitation in the future.

Albeit use of RCMs adds an extra tier of complexity to model fine-scale processes and phenomena relevant for climate change impact assessment at the local level hence with a probably reduced bias, it introduces a further dimension of uncertainty. Our results indicate that the total uncertainty in climate change signals of mean and extreme precipitation vary with seasons, with summer having the largest uncertainty. Similarly, the relative contributions of GCMs, RCMs and RCPs to the total uncertainty are consistent for mean and extreme precipitation, but vary with seasons. The boundary forcing of GCMs makes the greatest contribution to uncertainty in all seasons except for summer when RCMs play the most important role. Because the WAS-CORDEX RCMs (as for other CORDEX ensembles) are nested in a limited number of the CMIP5 GCMs, the obtained uncertainty is expected to be only a fraction of the total possible uncertainty and including more CMIP5 GCMs in the dynamical downscaling framework of the WAS-CORDEX RCMs may expand the uncertainty range. In addition, the scenario uncertainty may be underestimated since the extreme low emission scenario of RCM2.6 is not included in the CORDEX RCM projections.

Comparative analysis between the results of the WAS-CORDEX RCMs and the driving CMIP5 GCMs show the largest discrepancy for summer when model spatial revolution plays a more important role for precipitation simulations. This is indeed due to the diminishing influence of large-scale phenomena and a greater dependence of extreme precipitation on local processes in summer. In addition, the heavier the precipitation is, the larger the RCM-GCM results discrepancy will be. This underscores the urgent need of enlarging multi-model ensembles of CORDEX RCM integrations for local climate change impact analyses especially for summer when some extreme precipitation intensities may be totally missing in GCM simulations, leading, in turn, to an underestimation of the future extreme precipitation and flood hazards derived from GCM results.

## Methods

### Climate model data

The RCM ensemble within the Coordinated Regional Climate Downscaling Experiment (CORDEX) framework over the South Asia domain (WAS-CORDEX), known as “Region 6”, is used. The study domain is the extended Middle East region which includes 28 countries with a population exceeding 850 million people: Afghanistan, Armenia, Azerbaijan, Bahrain, Cyprus, Djibouti, Egypt, Eritrea, Ethiopia, Georgia, Iran, Iraq, Israel, Jordan, Kuwait, Lebanon, Oman, Pakistan, Palestinian, Qatar, Saudi Arabia, Somalia, Sudan, Syria, Turkey, Turkmenistan, United Arab Emirates and Yemen.

The WAS-CORDEX ensemble consists of 14 members: two RCMs, two greenhouse gas scenarios and six driving GCMs. The RCMs are (1) the Sveriges Meteorologiska och Hydrologiska institute (SMHI) Rossby Centre Regional Atmospheric Climate Model, version 4 (RCA4) and (2) the Max Planck Institute Regional Model (REMO). These WAS-CORDEX RCMs were used to downscale the results of six CMIP5 GCMs of CNRM-CM5, EC-EARTH, GFDL-ESM2M, IPSL-CM5A-MR, MIROC5 and MPI-ESM-LR. The attributes of RCMs and GCMs used in this study are presented in the Supplementary Table S1. All WAS-CORDEX RCM simulations performed at a model grid resolution of 0.44° × 0.44° are available for two Representative Concentration Pathways (RCP4.5 & RCP8.5). RCP4.5 and RCP8.5 represent mid and high levels of emission scenario respectively, corresponding to a radiative forcing of approximately 4.5 and 8.5 W/m^2^ by 2100^45^. The period 1971–2000 is considered for the current climate, while the late 21st century time slice of 2070–2099 is used for the future climate.

### Climate change signals

Climate change impact analysis is performed for mean and extreme precipitation over the Middle East region. For extreme precipitation, we focus on extreme events with different severities from moderate to rare. Albeit climate model results are more robust for the moderate extreme events, the rare extreme events are more relevant for flood studies. The moderate event is defined as extreme precipitation with 1-year return period (occurrence frequency of once per year). The extreme precipitation with 5- and 15-year return periods are referred to as very extreme event and rare extreme event, respectively. The return period for an empirical distribution is defined as the ratio of the length of the study period to the rank of precipitation values (with rank 1 corresponding to the largest value). The climate change signals are calculated as the ratio of daily extreme precipitation of a given return period for the future time period (2070–2099) to daily extreme precipitation of the same return period for the historical period (1971–2000). Similarly, the signals for mean precipitation are obtained by comparing the climatological mean values of the future and historical time periods. In order to compare the signals between different seasons, all the precipitation indicators are calculated separately for winter (December-January-February: DJF), spring (March-April-May: MAM), summer (June-July-August: JJA) and autumn (September-October-November: SON).

To measure the robustness of the climate change signals, a variety of methods have been proposed based on either the significance of changes relative to internal variability or the model agreement on the sign of change^2^. Although there is a high similarity between the results of different methods^46,47^, the latter is used here because, in some cases, climate change signals may be small compared to internal variability (i.e., statistically insignificant); however, the models agree on the direction of change which may still contain useful information for stakeholders and policymakers for developing adaptation and mitigation strategies^48^. We consider a climate change signal to be robust where at least 70% of the models agree on the direction of change. Otherwise, the change is interpreted as ‘unreliable’ and masked in gray color on the maps.

### Uncertainty analysis

Although combining climate change results from different members of a model ensemble into a single projection (i.e., mean ensemble) presents a user-friendly information for policymakers and stakeholders, it conceals the uncertainty in the ensemble projections. The WAS-CORDEX RCM ensemble used here includes two RCMs forced by six CMIP5 GCMs and two emission scenarios (RCPs). The total uncertainty in mean and extreme precipitation changes is the sum of the RCM uncertainty arising from the WAS-CORDEX RCMs, the GCM uncertainty arising from the driving CMIP5 GCMs and the scenario uncertainty (referred to as RCP uncertainty in this paper) arising from the forcing scenarios RCP4.5 and RCP8.5. As the larger sample size of the GCMs compared to the RCMs and the RCPs can affect the amount of the GCM uncertainty, the variance decomposition-same sample size (VD-SSS) approach developed by Hosseinzadehtalaei *et al*.^40^ is used for GCM uncertainty quantification. Following the VD-SSS approach, first the multi-model medians of the climate change signals of the WAS-CORDEX ensemble regarding each GCM are computed. Afterwards, two (common sample size among the uncertainty components in our case) GCMs are randomly sampled from the GCM population (six GCMs in our case) in the framework of a bootstrapping technique and the standard deviation between the multi-model medians of the two sampled GCMs is calculated. This procedure is repeated a large number of times and the median of the standard deviations represents the GCM uncertainty. For the RCM and RCP uncertainty, we use the conventional variance decomposition (VD) method. The RCM (RCP) uncertainty is computed as the standard deviation between the multi-model median of the climate change signals of the WAS-CORDEX ensemble following the REMO2009 RCM (RCP4.5) and that following the RCA4 RCM (RCP8.5).

### Downscaling signals

The ensemble downscaling signal (EDS) of the WAS-CORDEX RCMs is calculated in a similar procedure to the downscaling signal (DS) method developed by Giorgi *et al*.^49^. The spatial anomaly of extreme precipitation changes derived from each RCM run is first calculated by subtracting the regional median of the changes over the study domain from the change in each model grid cell. Similarly, the spatial anomaly of the changes is determined for the corresponding driving GCMs. The difference between the spatial anomaly of RCM-based changes and that of the corresponding driving GCM-based changes is then obtained. The EDS is finally calculated by taking a median over the different RCM-GCM combinations. The mathematical expression of the method is as follows:

1 $$EDS={{\rm{median}}}_{{i}}[({\rm{\Delta }}{P}_{RC{M}_{i,s,T}}-\langle {\rm{\Delta }}{P}_{RC{M}_{i,s,T}}\rangle )-({\rm{\Delta }}{P}_{GC{M}_{i,s,T}}-\langle {\rm{\Delta }}{P}_{GC{M}_{i,s,T}}\rangle )]$$

where ∆P is the extreme precipitation change for a given return period *T* and season *s*, $\langle {\rm{\Delta }}P\rangle$ is the median (instead of mean to exclude the possible outliers effect) of changes for a given RCM or GCM over the study domain for the same return period and season, *i* denotes each RCM-GCM combination in the WAS-CORDEX ensemble and median_*i*_ means the median of downscaling signals over *i* notation. The median of downscaling signals reduces the random noise stemmed from natural variability.

Because RCMs and GCMs use different horizontal resolutions, we regridded all the original extreme precipitation changes to the same resolution. The results of several interpolation methods such as linear, nearest neighbor, cubic, cubic spline were carefully compared to investigate the sensitivity of the results to the interpolation method used. The comparison shows that the choice of interpolation method does not influence the main conclusions of the EDS analysis.

### Data availability

The CMIP5 GCM and CORDEX RCM data are freely available at the website of the Earth System Grid Federation (https://esgf-index1.ceda.ac.uk).

## Electronic supplementary material

Supplementary information
