# Supplementary material for: Seasonally varying footprint of climate change on precipitation in the Middle East
Source: Sci Rep. 2018 Mar 13;8:4435. doi: 10.1038/s41598-018-22795-8 (PMC5849753; doi:10.1038/s41598-018-22795-8)
Supplement: Supplementary file 1 — Supplementary information [file 41598_2018_22795_MOESM1_ESM.docx]

**Supplementary information**

**Seasonally varying footprint of climate change on precipitation in the Middle East**

Hossein Tabari^1^* and Patrick Willems^1,2^

1) Hydraulics Division, Department of Civil Engineering, KU Leuven, Kasteelpark Arenberg 40, BE-3001 Leuven, Belgium.

2) Department of Hydrology and Hydraulic Engineering, Vrije Universiteit Brussel, Belgium.

*Corresponding author: e-mail: hossein.tabari@kuleuven.be; [tabari.ho@gmail.com](mailto:tabari.ho@gmail.com), Tel: +32 16 377007, Fax: +32 16 321989.

**Text S1. Comparison between uncertainty results of VD and VD-SSS approaches**

In order to limit the effect of the larger sample size of the driving GCMs compared to the sample size of the RCMs and RCPs (6 GCMs versus 2 RCPs and 2 RCMs) on the GCM uncertainty rate, the variance decomposition-same sample size (VD-SSS; Hosseinzadehtalaei et al.^1^) approach was used. Figure S1 shows a comparison between the GCM uncertainty obtained from the conventional variance decomposition (VD) and VD-SSS methods for extreme and mean precipitation in different seasons. As one can see, the conventional VD method overestimates the GCM uncertainty for both extreme and mean precipitation for all seasons. The overestimations range from 5.6% for mean summer precipitation to 12% for extreme precipitation of 1-year return period for the same season. The VD-SSS effectively removes that part of the GCM uncertainty which is because of the larger sample size. Hence, the uncertainty amount calculated by the VD-SSS is only due to the variance among GCMs.

**
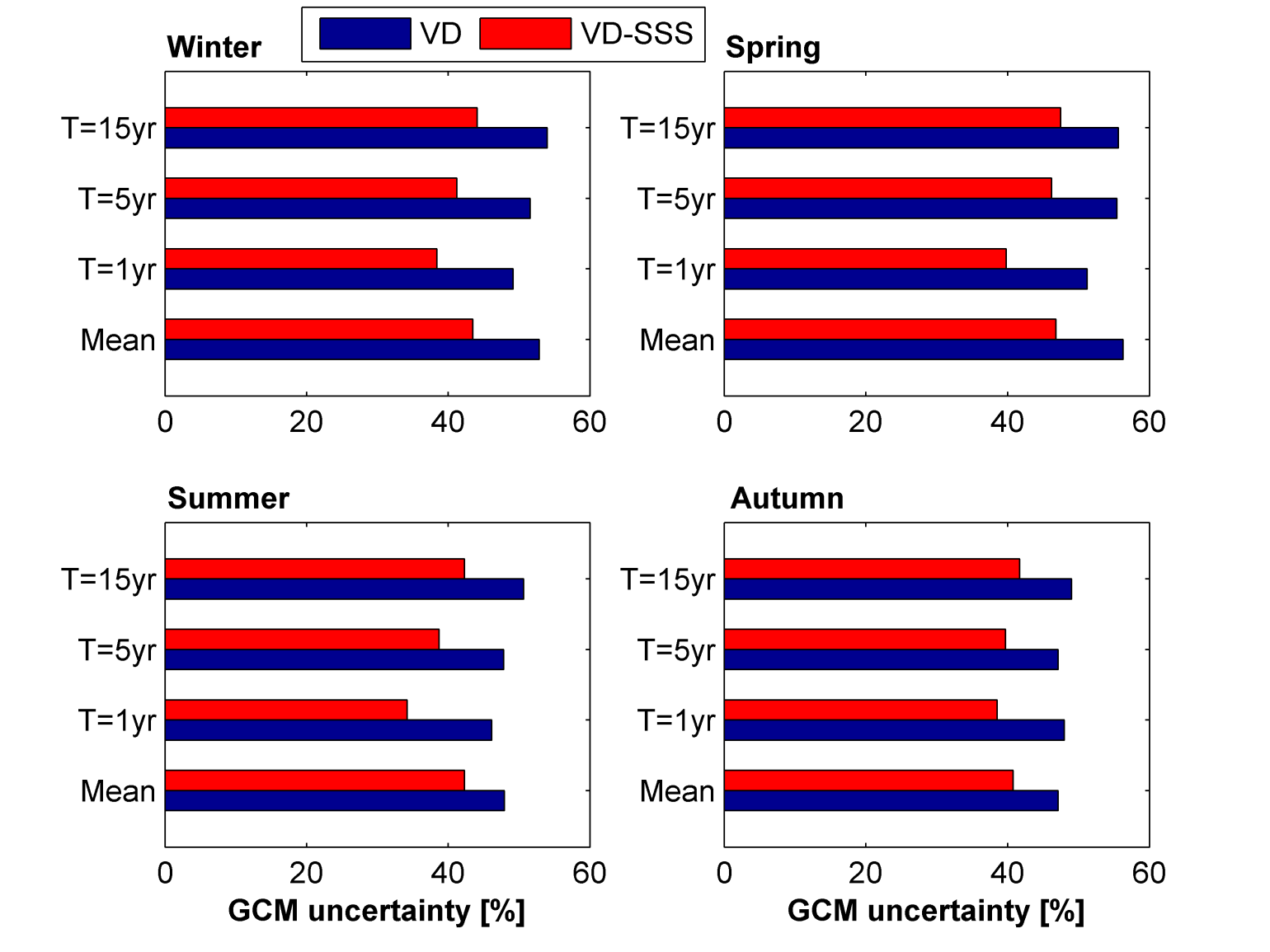
**

**Figure S1 | Comparison between GCM uncertainty obtained from variance decomposition (VD) and variance decomposition-same sample size (VD-SSS) methods for extreme and mean precipitation in different seasons**. T=15yr, T=5yr and T=1yr refer to extreme precipitation of 15-, 5- and 1-year return periods, respectively. ‘Mean’ corresponds to mean precipitation. Each bar of the plot corresponds to the median of the GCM uncertainty over the Middle East domain. The figure was created using the software MATLAB (version 2013a) <http://www.mathworks.com/products/matlab/>.


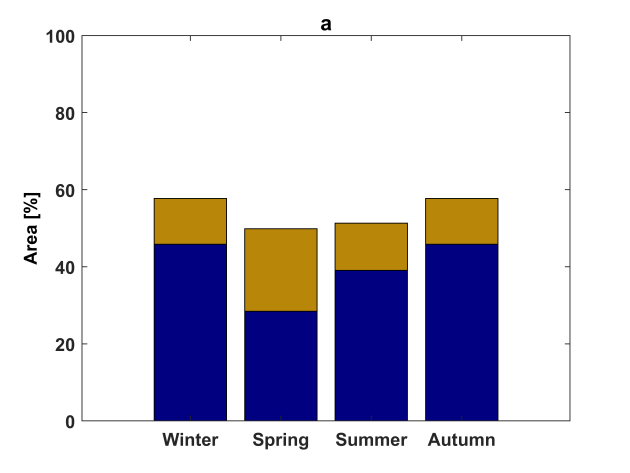

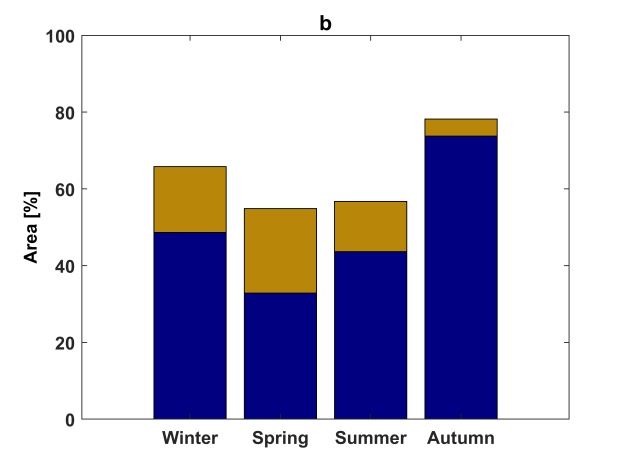

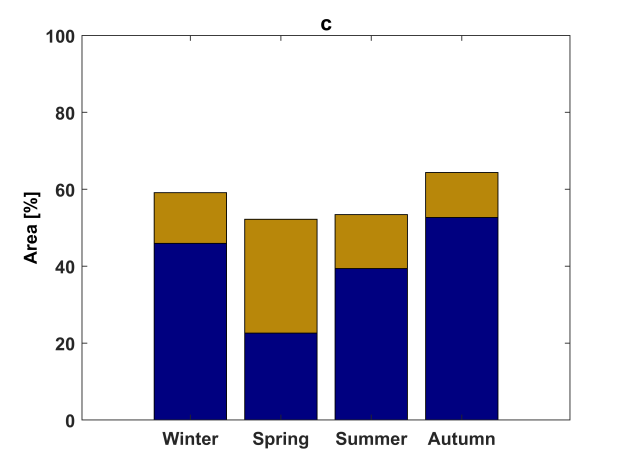

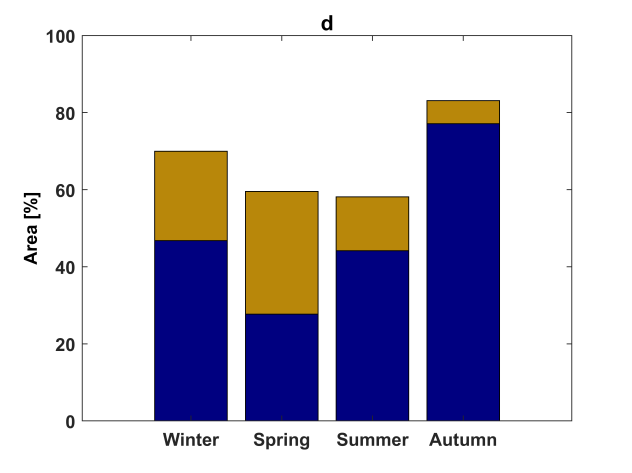

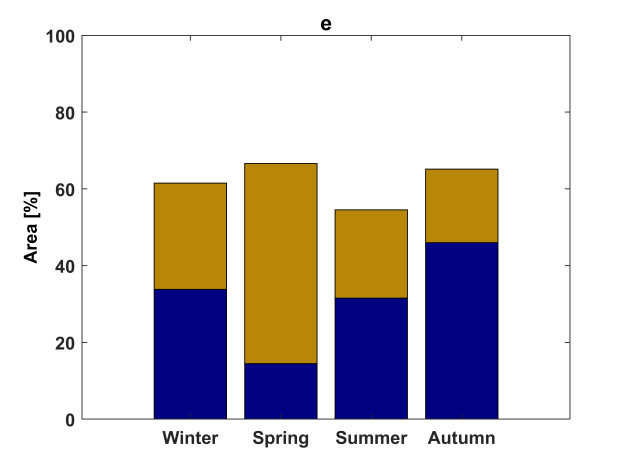

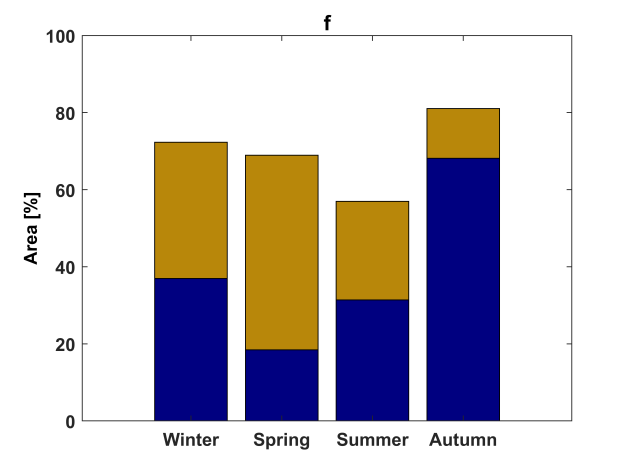

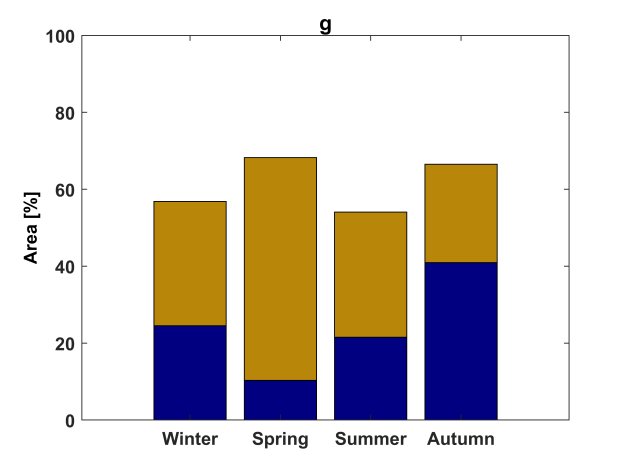

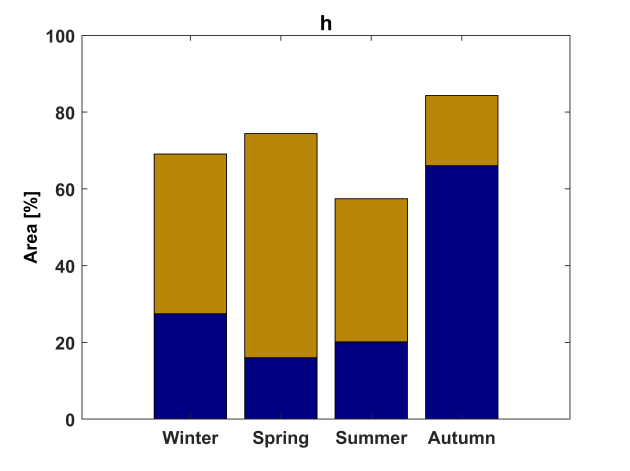


**Figure S2 | Percentage of the Middle East area with robust changes in mean (g, h) and extreme precipitation of (a, b) 15-, (c, d) 5- and (e, f) 1-year return periods for RCP4.5 (a, c, e, g) and RCP8.5 (b, d, f, h).** Blue and brown colors denote robust increase and decrease, respectively. The figure was created using the software MATLAB (version 2013a) <http://www.mathworks.com/products/matlab/>.

|  | **GCM uncertainty** | **RCM uncertainty** | **RCP uncertainty** |
| --- | --- | --- | --- |
| **15-yr** | 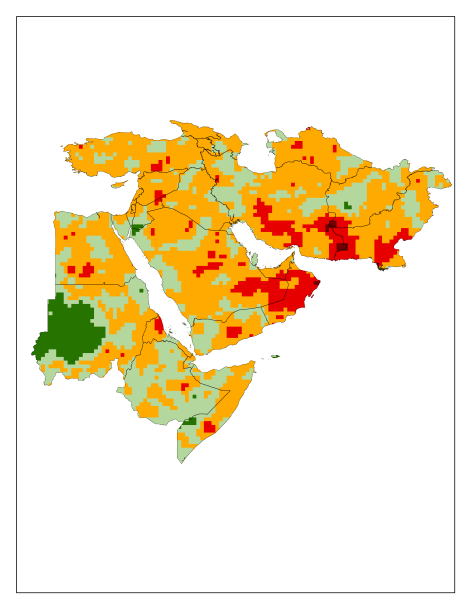 | 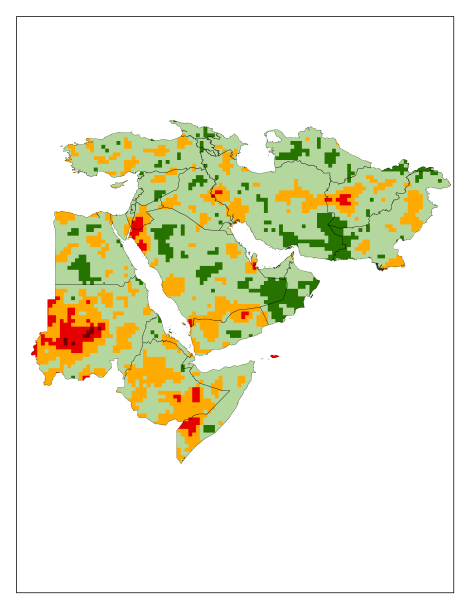 | 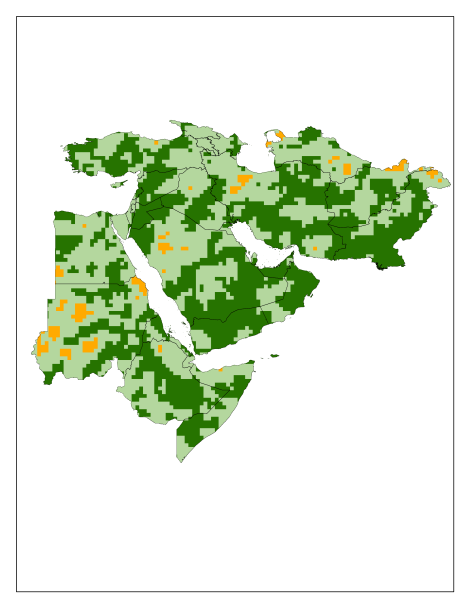 |
| **5-yr** | 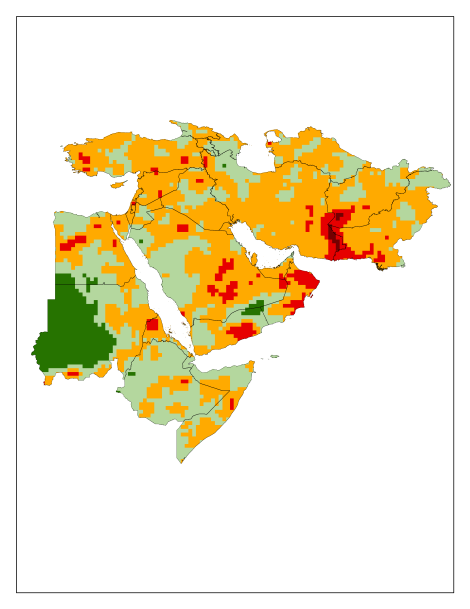 | 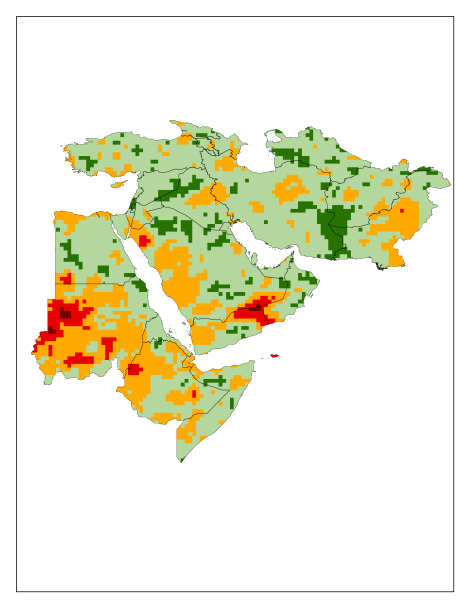 | 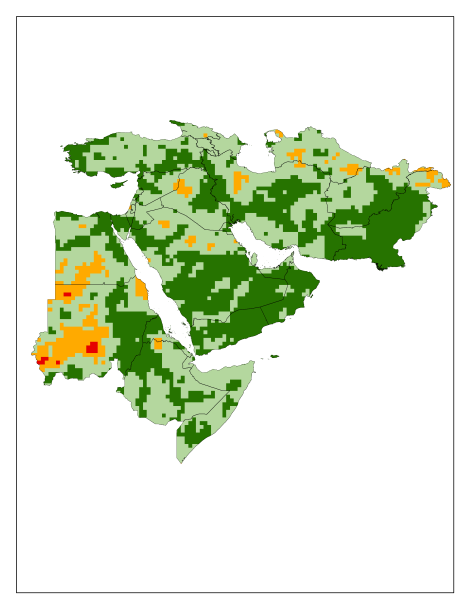 |
| **1-yr** | 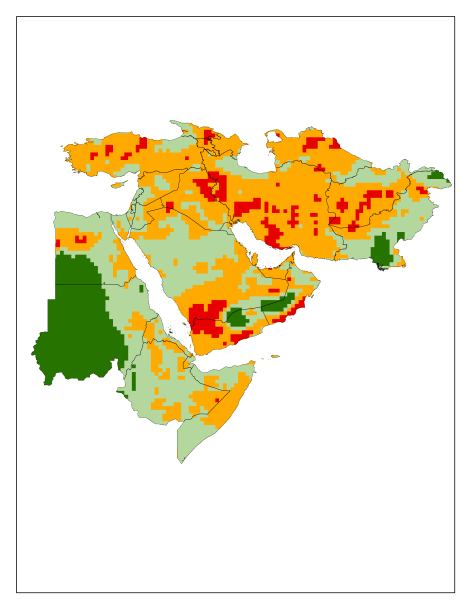 | 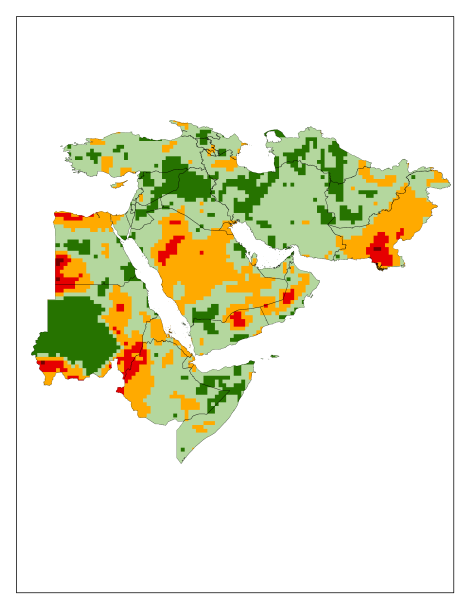 | 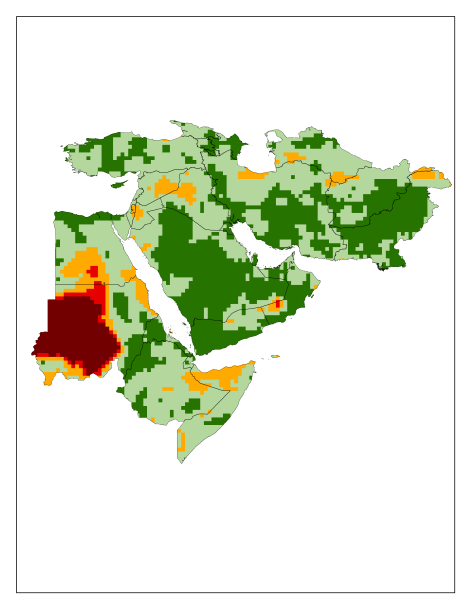 |
| **Mean** | 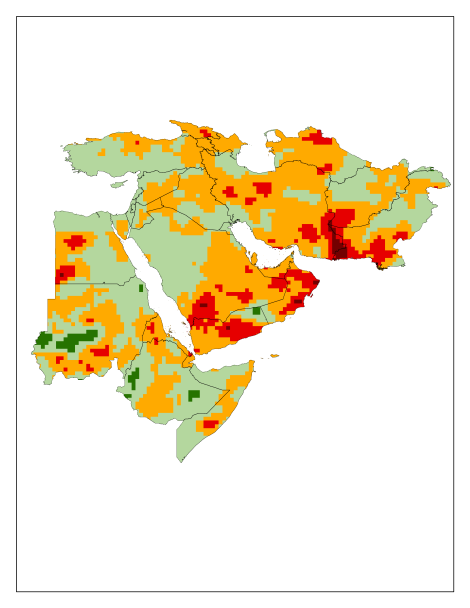 | 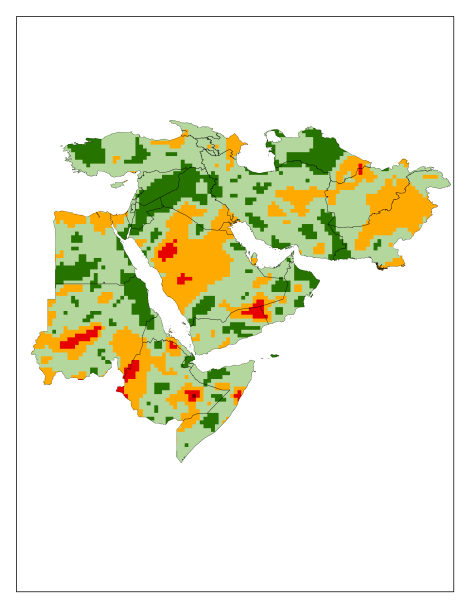 | 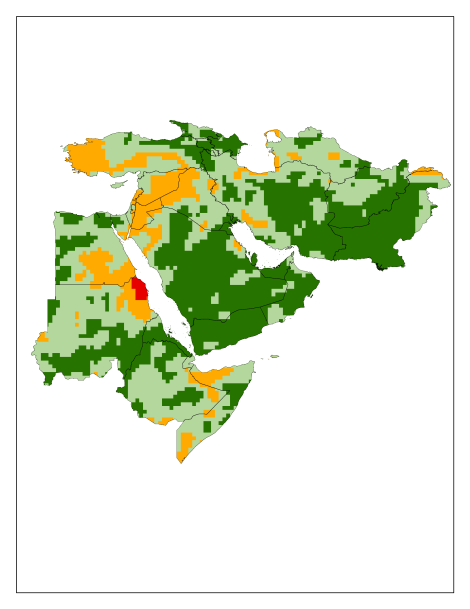 |
| 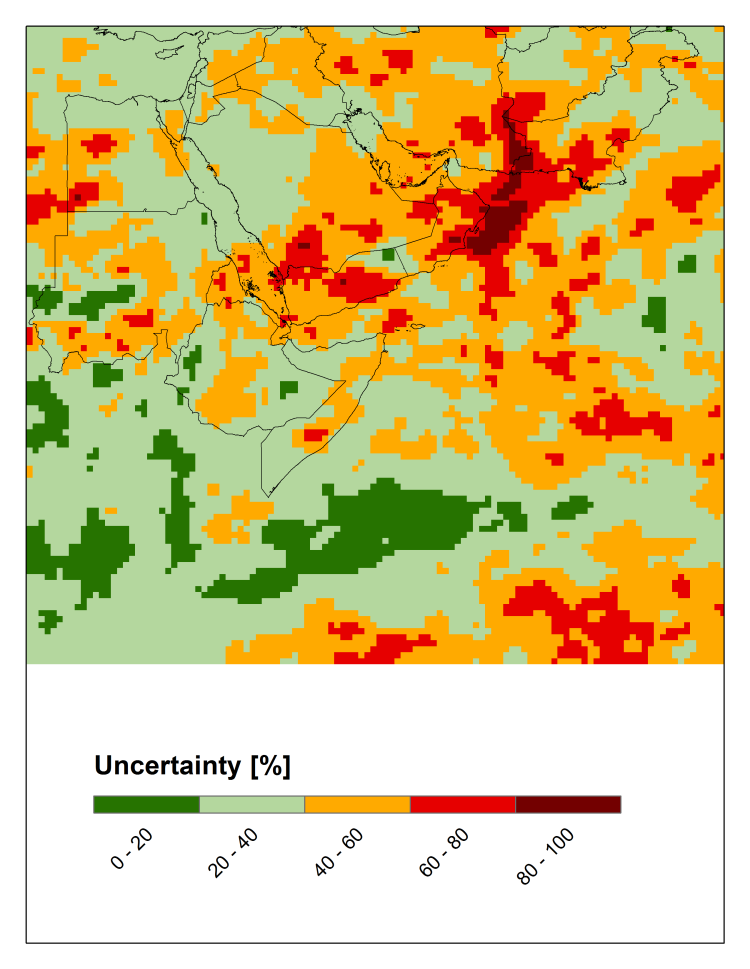 | | | |

**Figure S3 | The local fraction of total uncertainty in extreme and mean precipitation changes explained by GCM, RCM and RCP uncertainties over the Middle East region for the winter season.** T=15yr, T=5yr and T=1yr refer to extreme precipitation of 15-, 5- and 1-year return periods, respectively. ‘Mean’ corresponds to mean precipitation. The maps were generated using the software ArcGIS (version 10) <http://www.esri.com/products>.

|  | **GCM uncertainty** | **RCM uncertainty** | **RCP uncertainty** |
| --- | --- | --- | --- |
| **15-yr** | 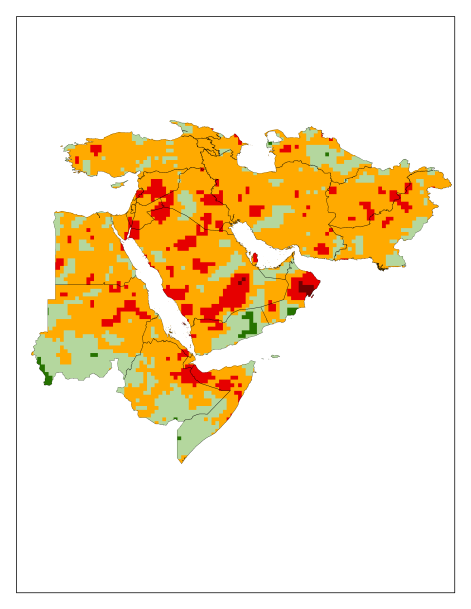 | 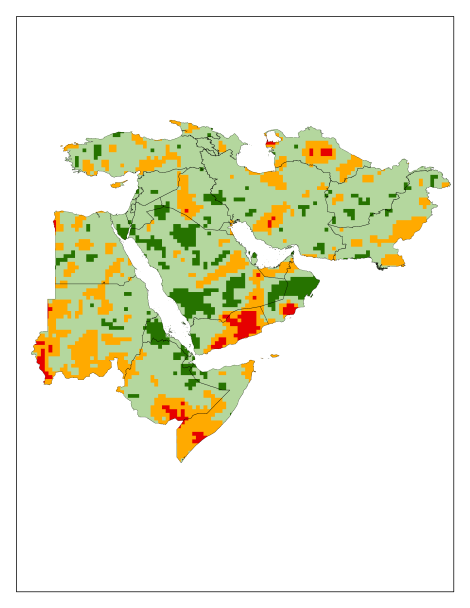 | 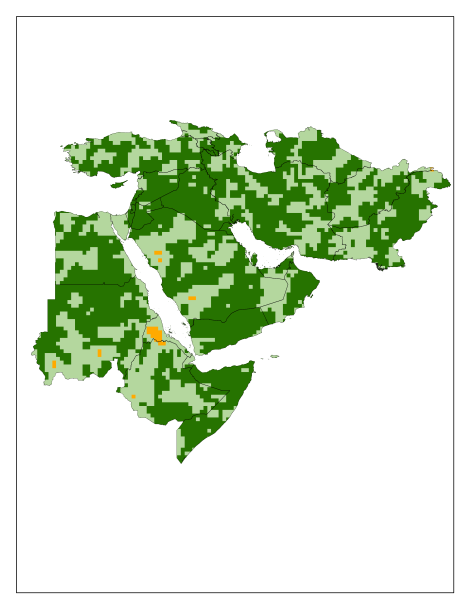 |
| **5-yr** | 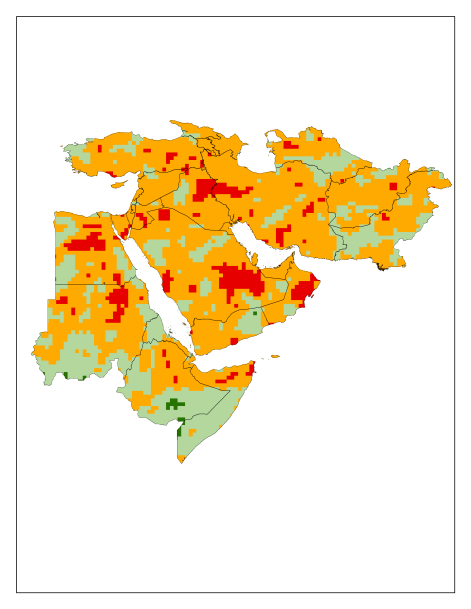 | 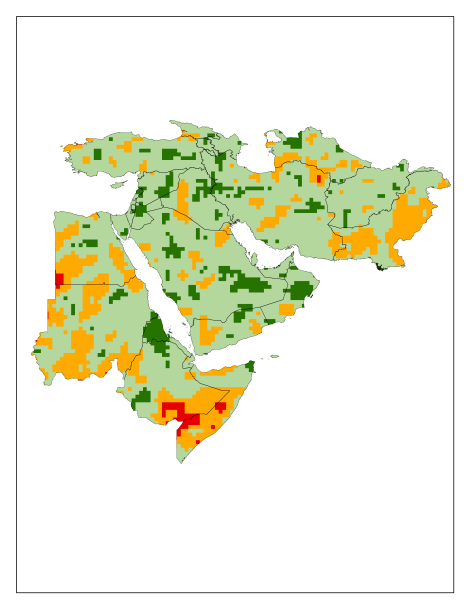 | 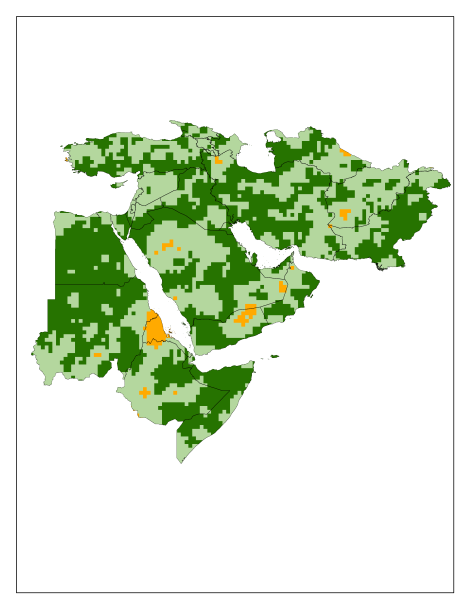 |
| **1-yr** | 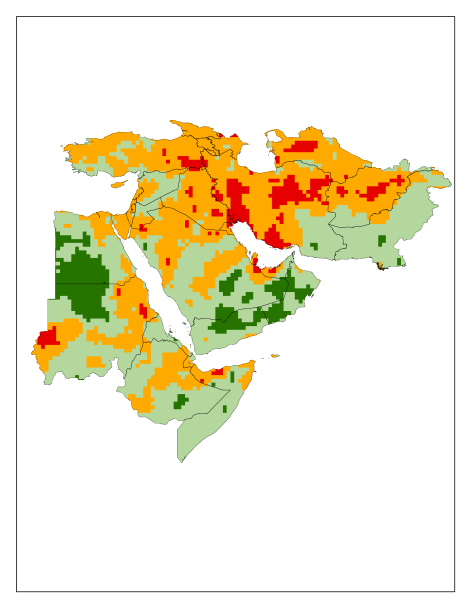 | 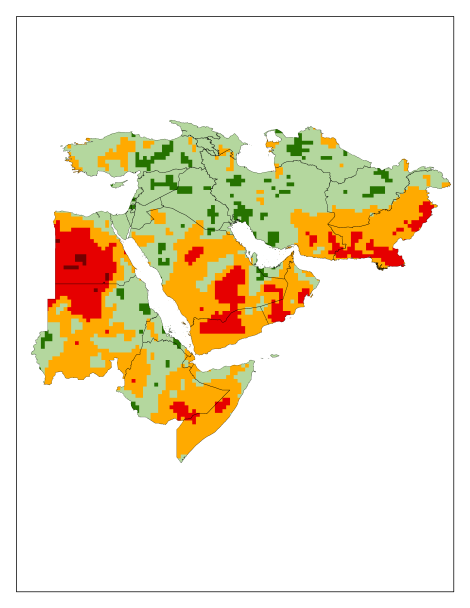 | 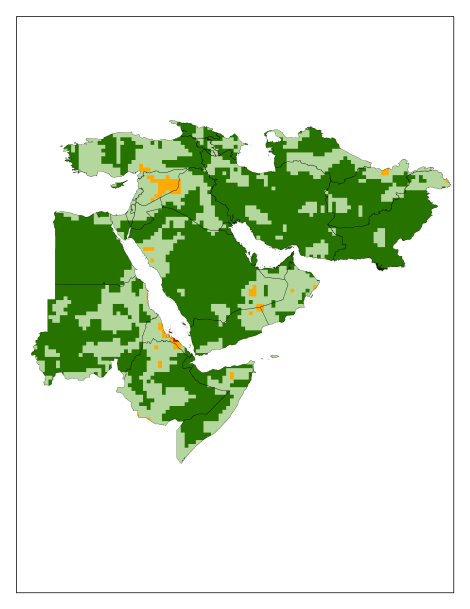 |
| **Mean** | 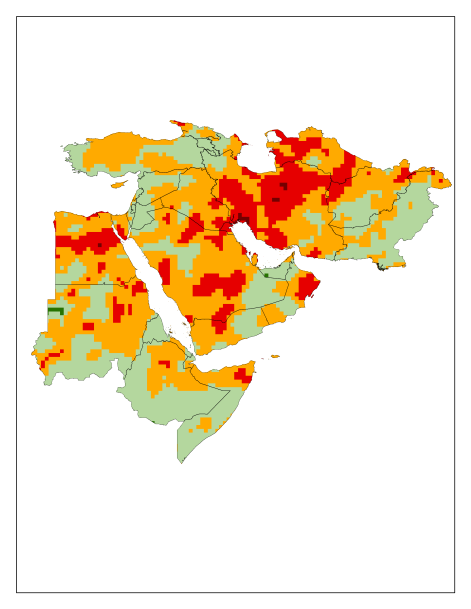 | 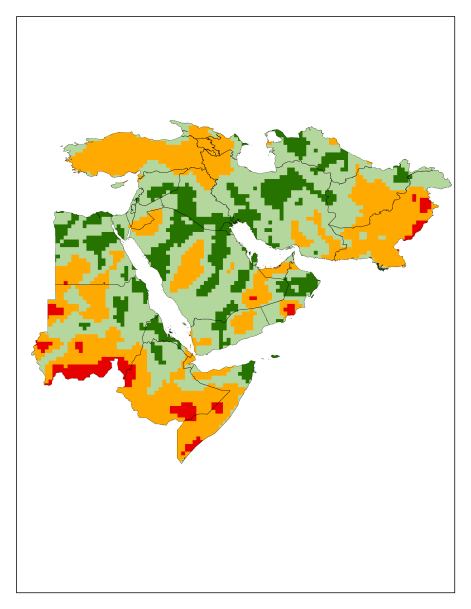 | 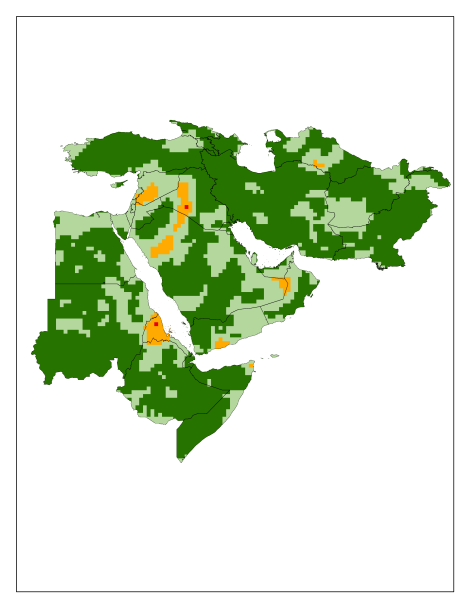 |
| 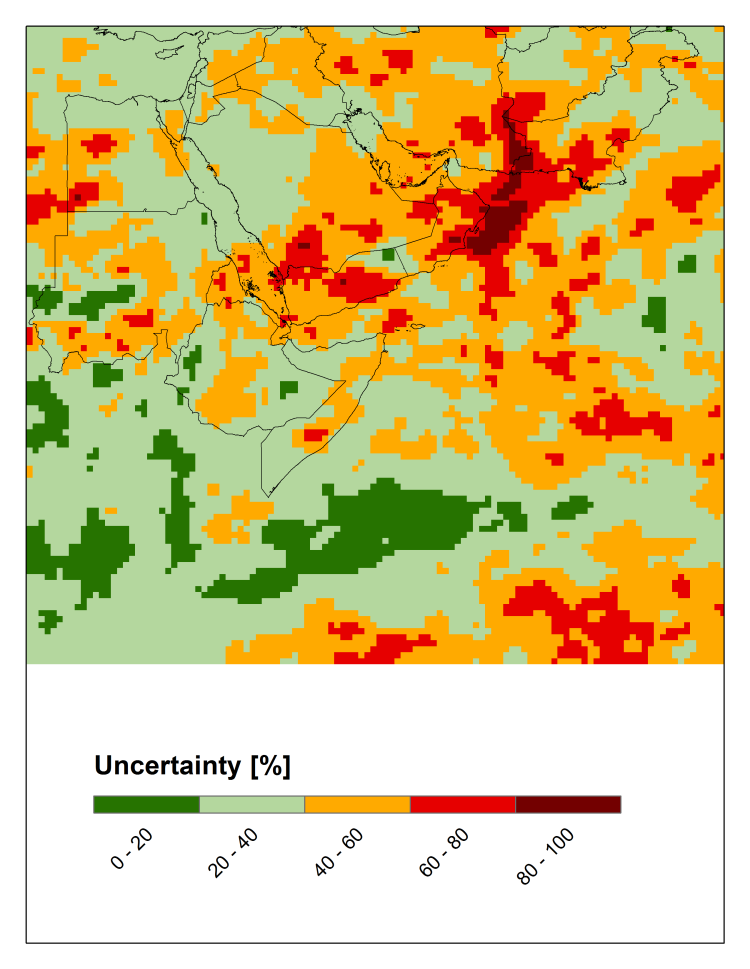 | | | |

**Figure S4 | The local fraction of total uncertainty in extreme and mean precipitation changes explained by GCM, RCM and RCP uncertainties over the Middle East region for the spring season.** T=15yr, T=5yr and T=1yr refer to extreme precipitation of 15-, 5- and 1-year return periods, respectively. ‘Mean’ corresponds to mean precipitation. The maps were generated using the software ArcGIS (version 10) <http://www.esri.com/products>.

|  | **GCM uncertainty** | **RCM uncertainty** | **RCP uncertainty** |
| --- | --- | --- | --- |
| **15-yr** | 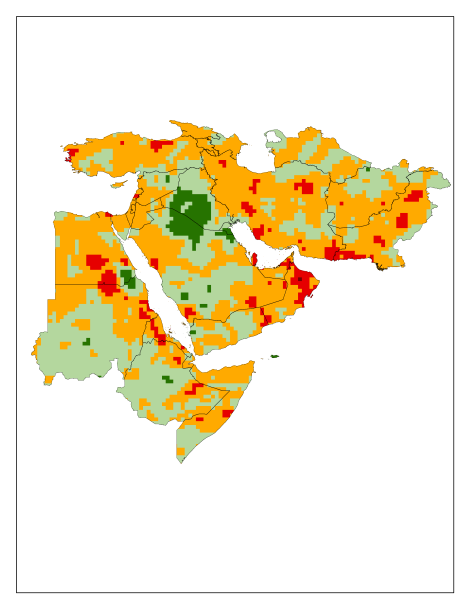 | 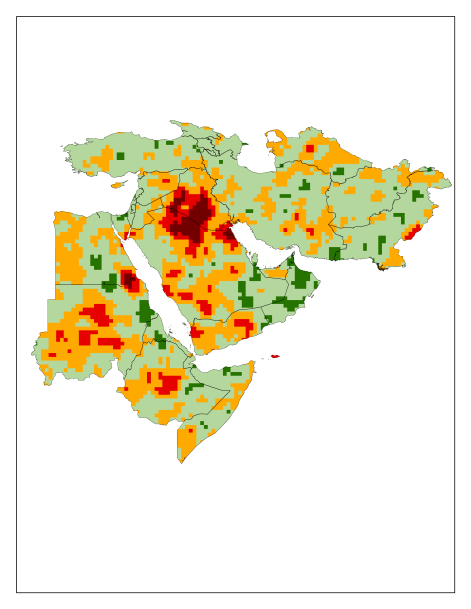 | 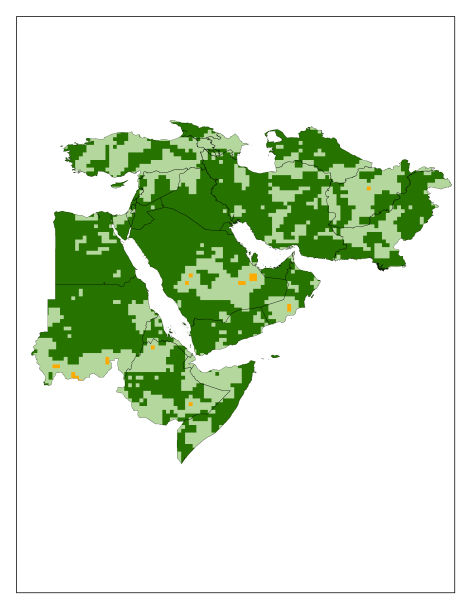 |
| **5-yr** | 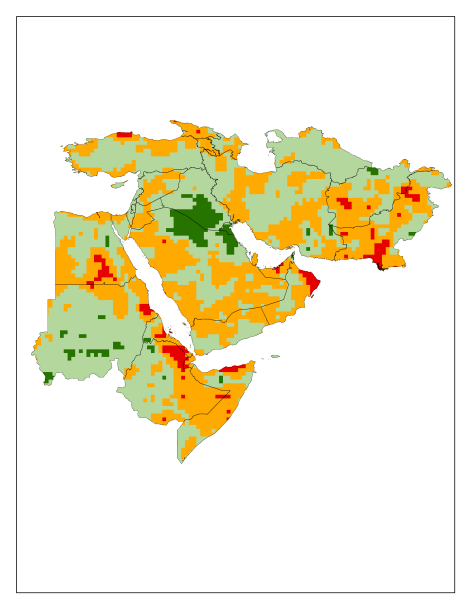 | 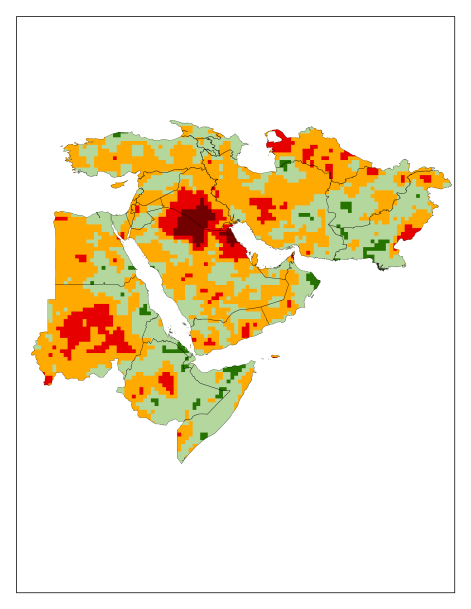 | 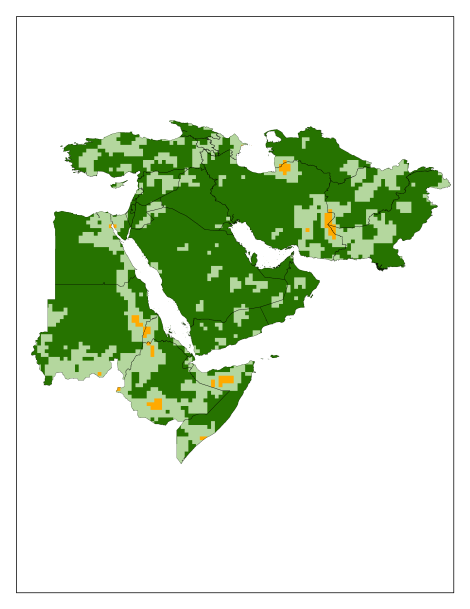 |
| **1-yr** | 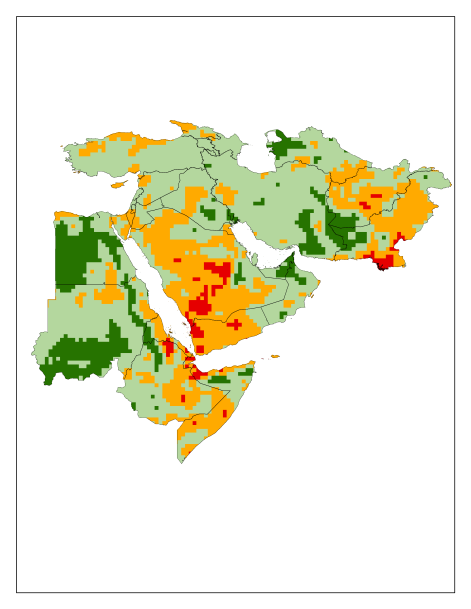 | 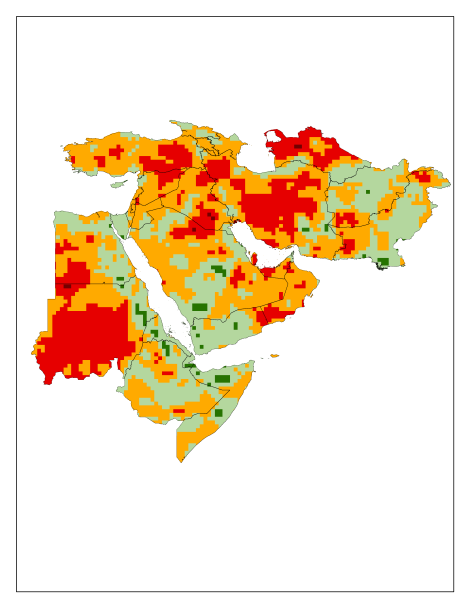 | 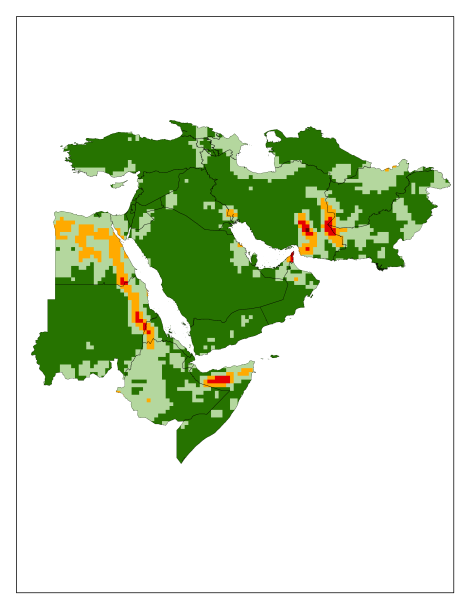 |
| **Mean** | 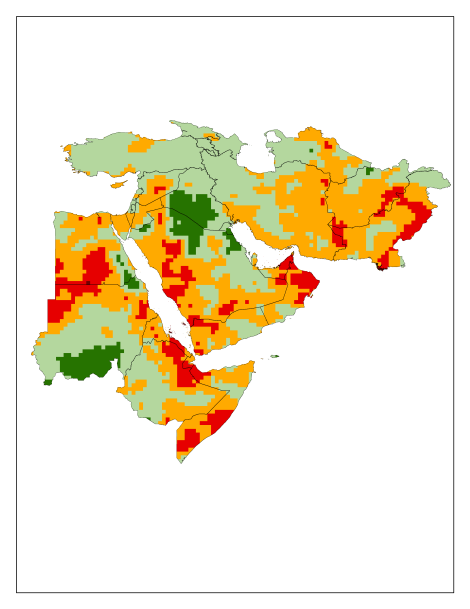 | 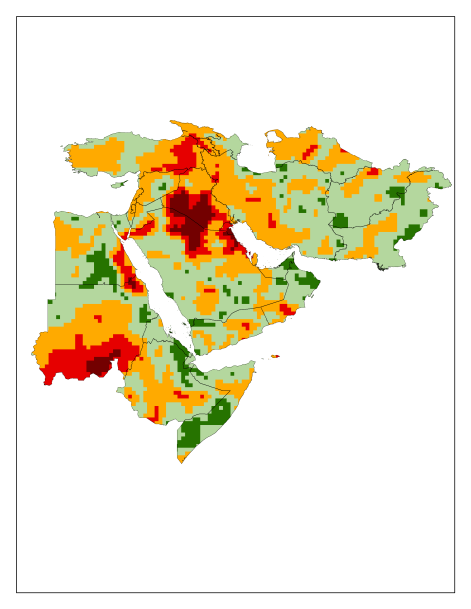 | 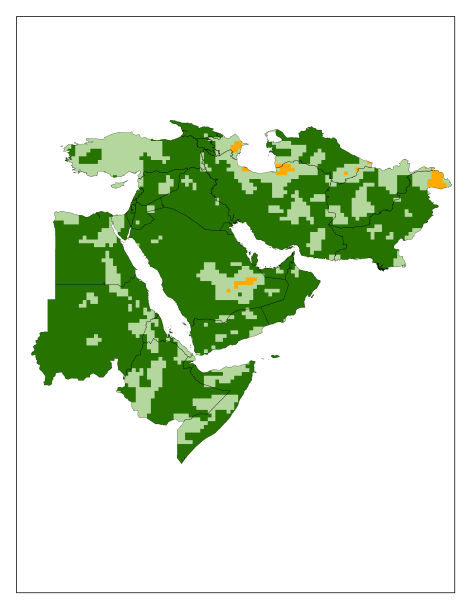 |
| 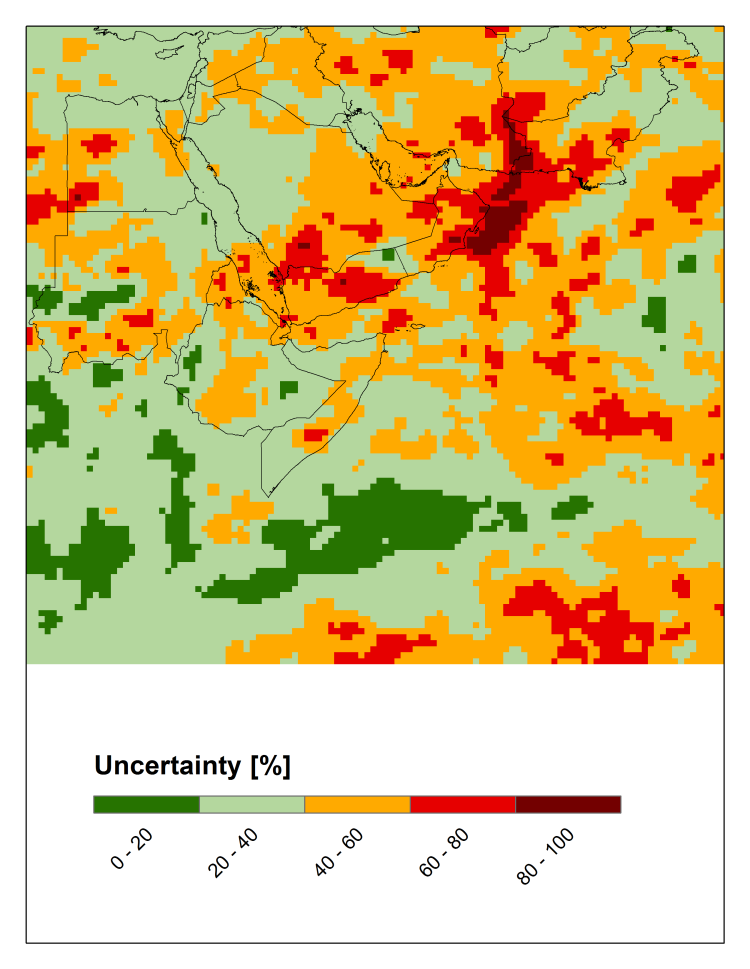 | | | |

**Figure S5 | The local fraction of total uncertainty in extreme and mean precipitation changes explained by GCM, RCM and RCP uncertainties over the Middle East region for the summer season.** T=15yr, T=5yr and T=1yr refer to extreme precipitation of 15-, 5- and 1-year return periods, respectively. ‘Mean’ corresponds to mean precipitation. The maps were generated using the software ArcGIS (version 10) <http://www.esri.com/products>.

|  | **GCM uncertainty** | **RCM uncertainty** | **RCP uncertainty** |
| --- | --- | --- | --- |
| **15-yr** | 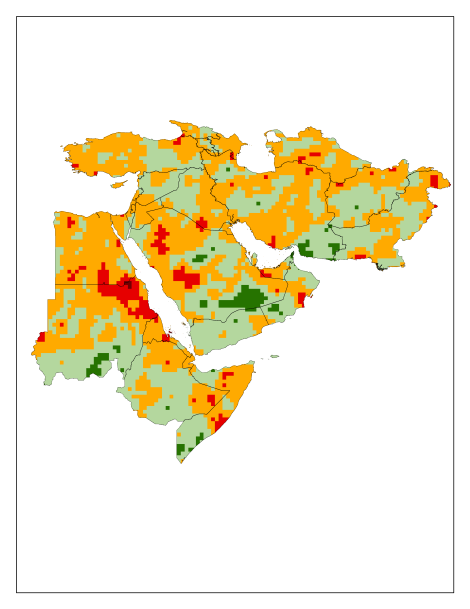 | 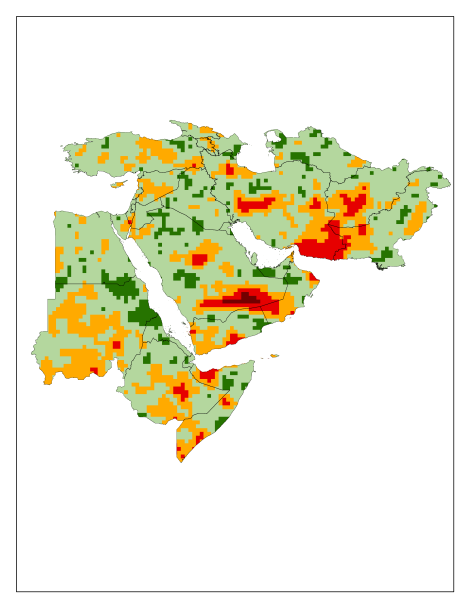 | 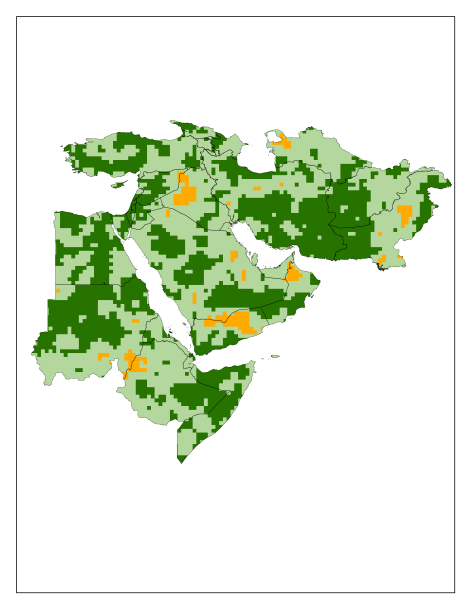 |
| **5-yr** | 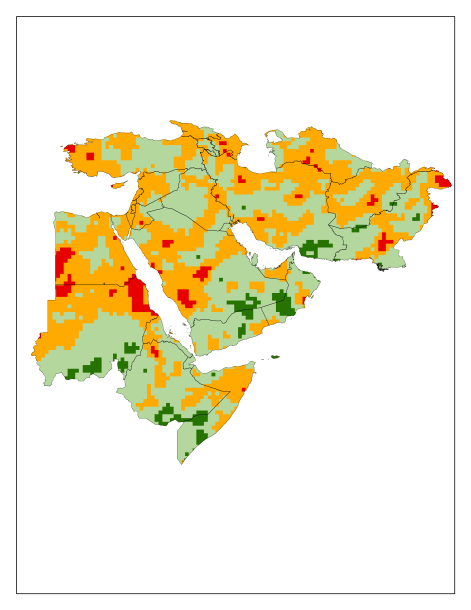 | 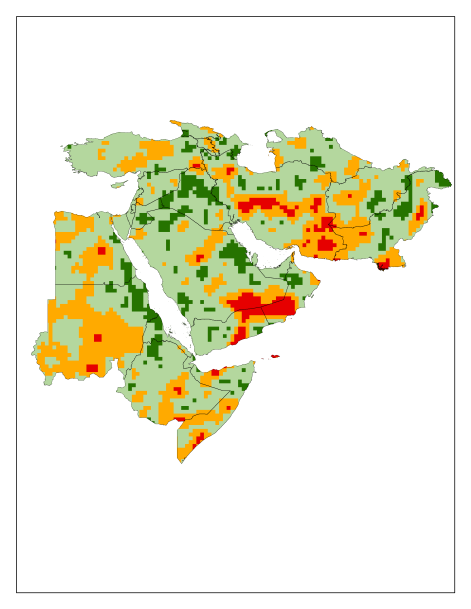 | 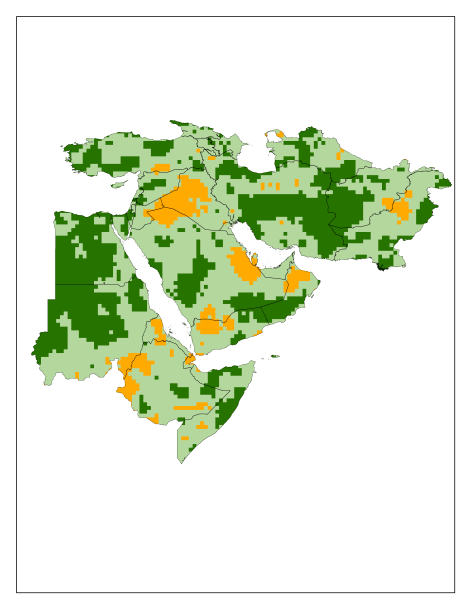 |
| **1-yr** | 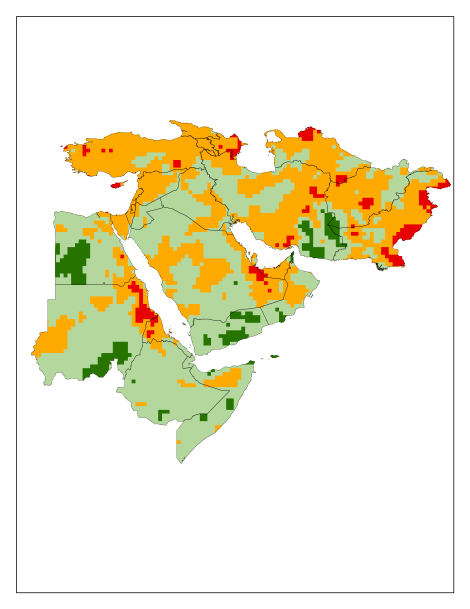 | 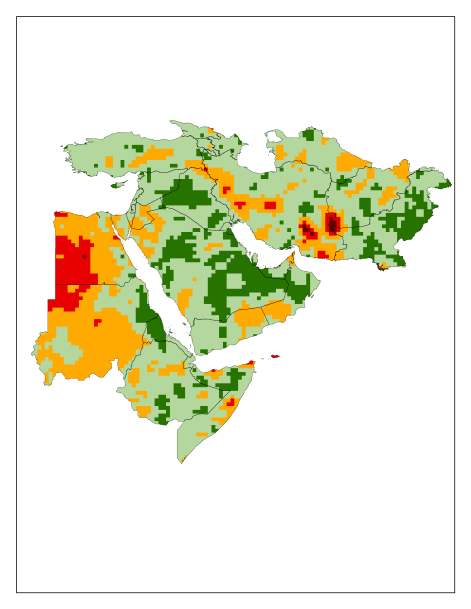 | 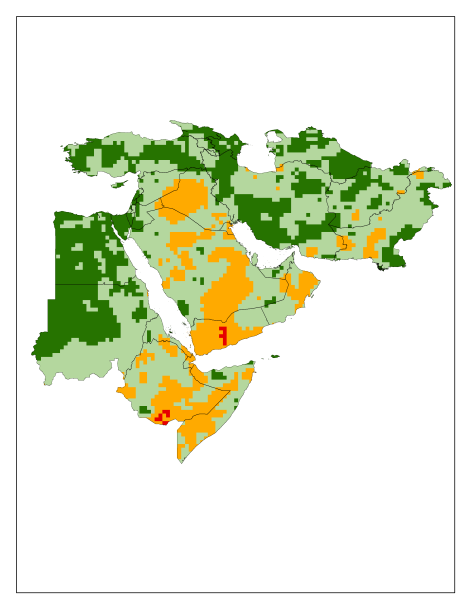 |
| **Mean** | 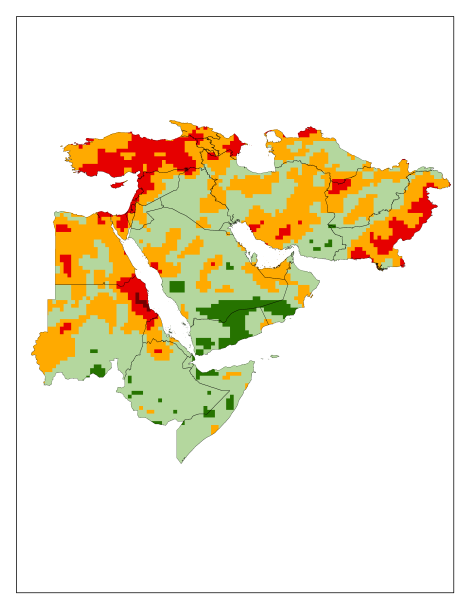 | 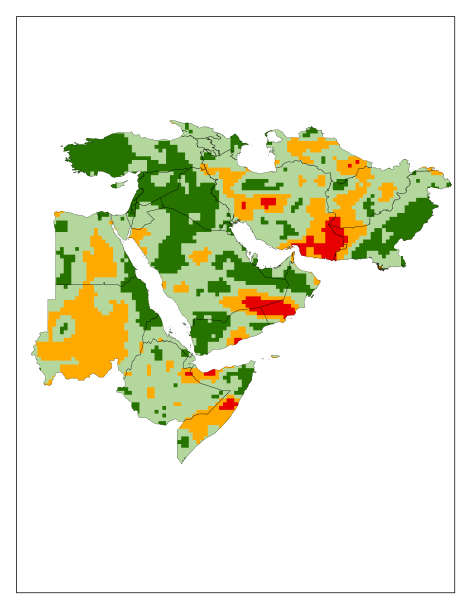 | 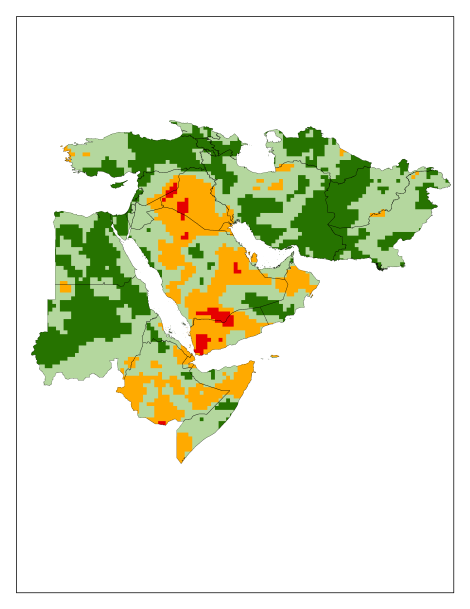 |
| 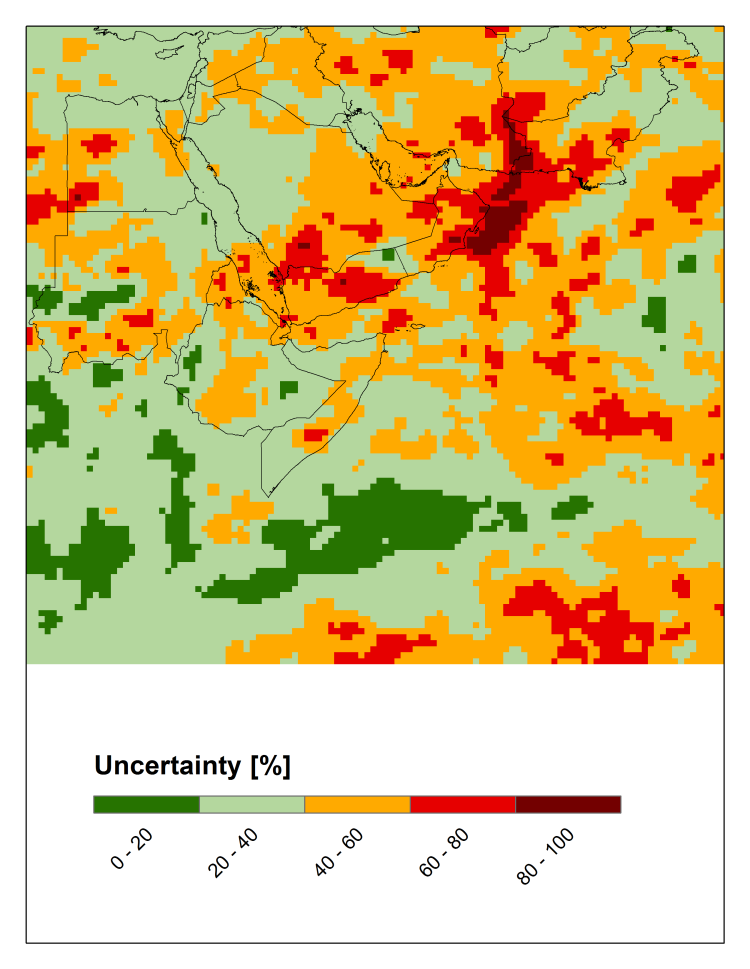 | | | |

**Figure S6 | The local fraction of total uncertainty in extreme and mean precipitation changes explained by GCM, RCM and RCP uncertainties over the Middle East region for the autumn season.** T=15yr, T=5yr and T=1yr refer to extreme precipitation of 15-, 5- and 1-year return periods, respectively. ‘Mean’ corresponds to mean precipitation. The maps were generated using the software ArcGIS (version 10) <http://www.esri.com/products>.

|  | **RCP4.5** | **RCP8.5** |
| --- | --- | --- |
| **15-yr** | **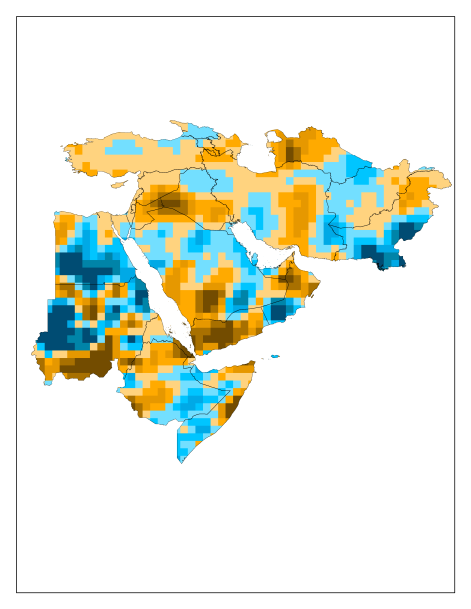** | **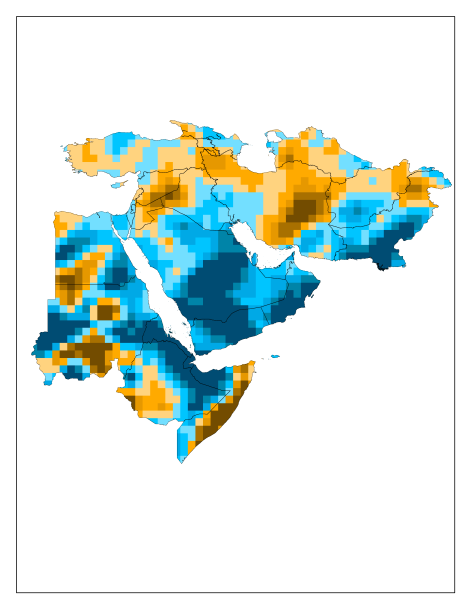** |
| **5-yr** | **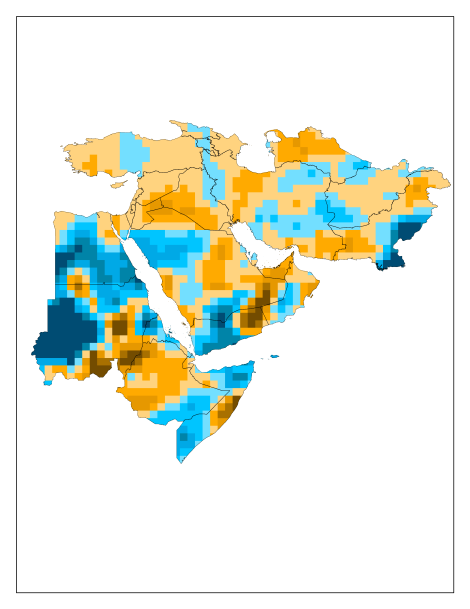** | **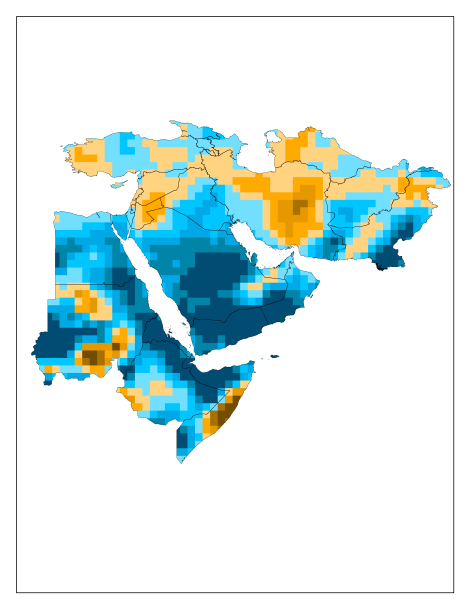** |
| **1-yr** | **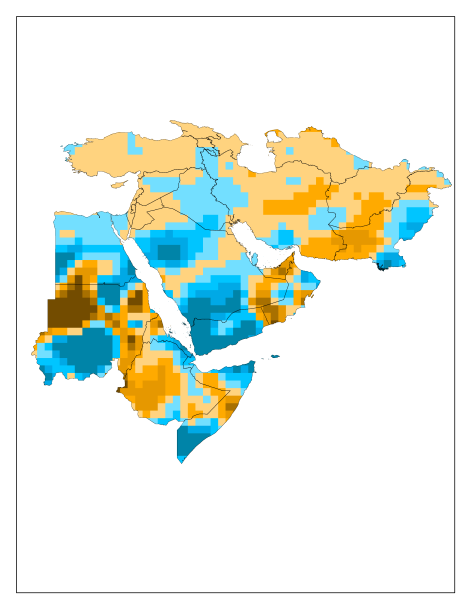** | **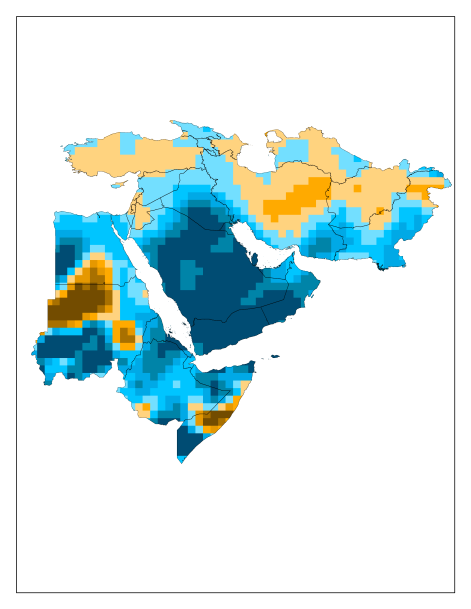** |
| **Mean** | **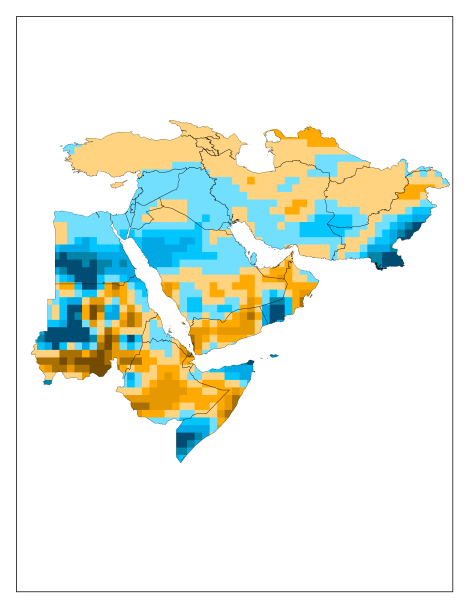** | **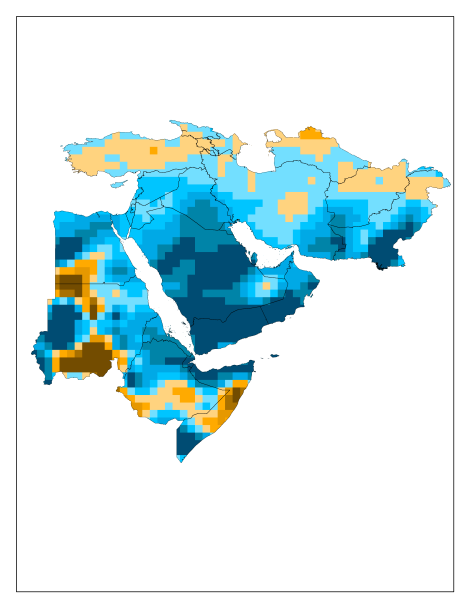** |
| 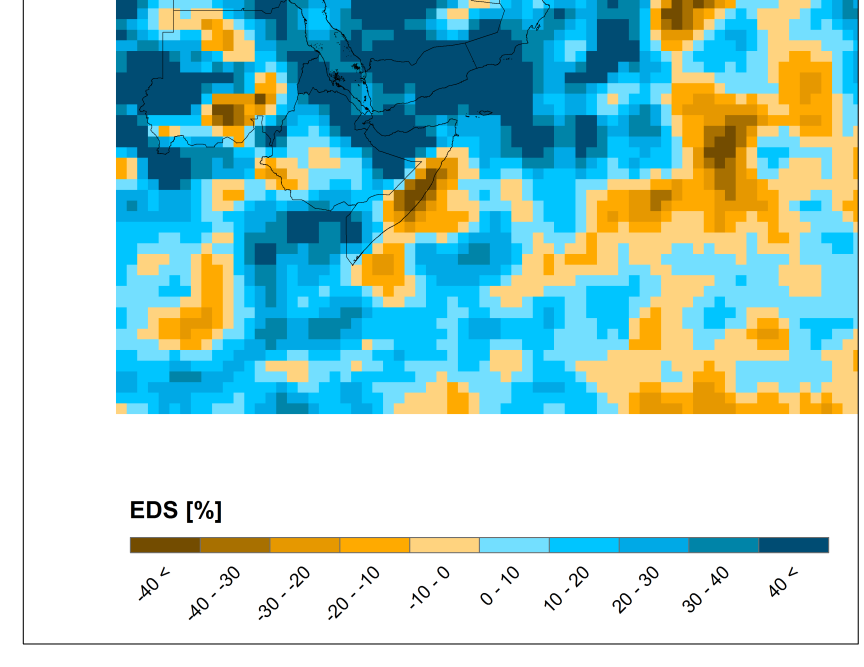 | | |

**Figure S7 | Ensemble downscaling signal of extreme and mean precipitation for RCP4.5 and RCP8.5 over the Middle East region for the winter season.** T=15yr, T=5yr and T=1yr refer to extreme precipitation of 15-, 5- and 1-year return periods, respectively. ‘Mean’ corresponds to mean precipitation. The maps were generated using the software ArcGIS (version 10) <http://www.esri.com/products>.

|  | **RCP4.5** | **RCP8.5** |
| --- | --- | --- |
| **15-yr** | 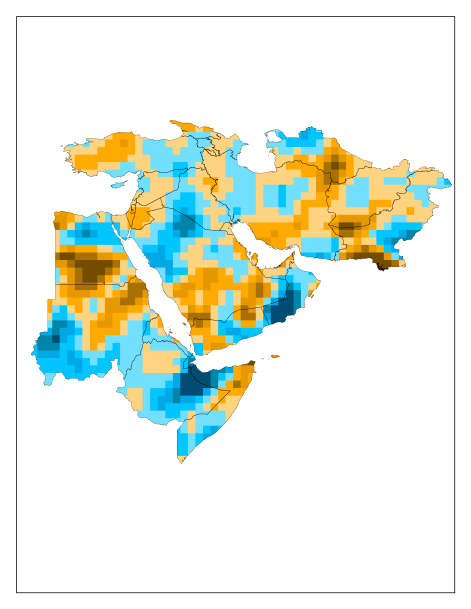 | 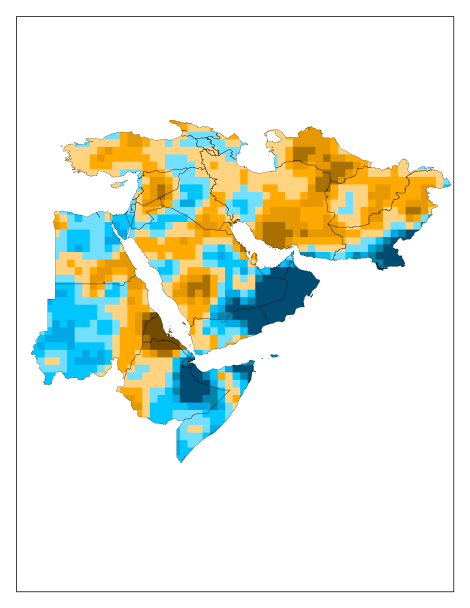 |
| **5-yr** | 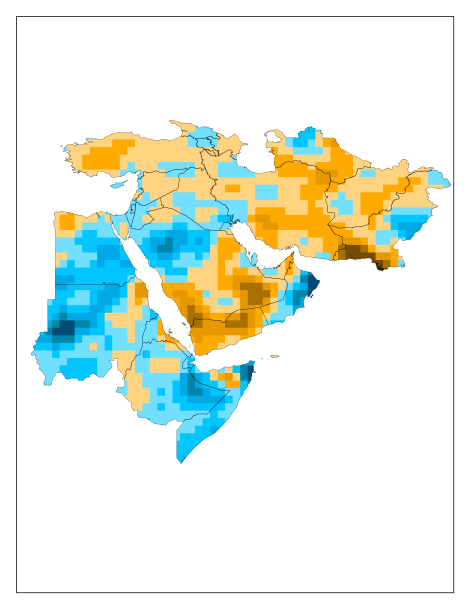 | 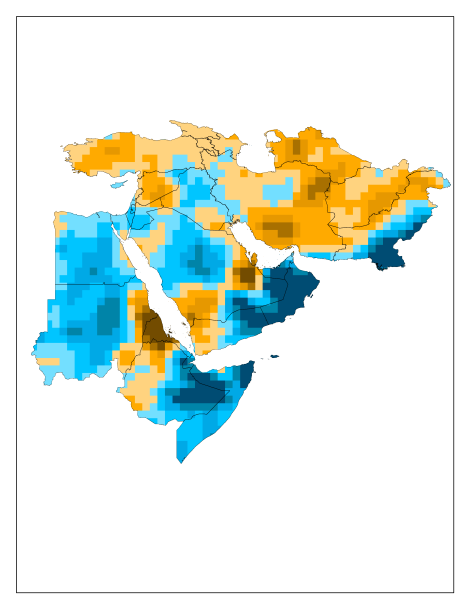 |
| **1-yr** | 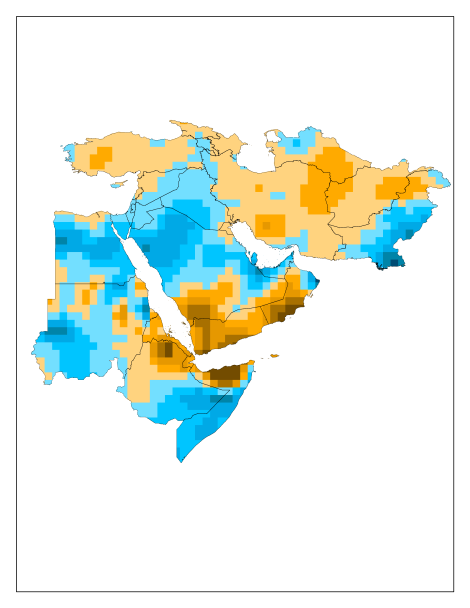 | 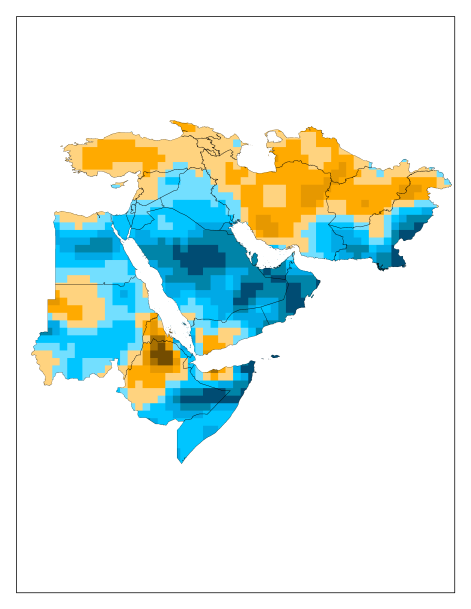 |
| **Mean** | 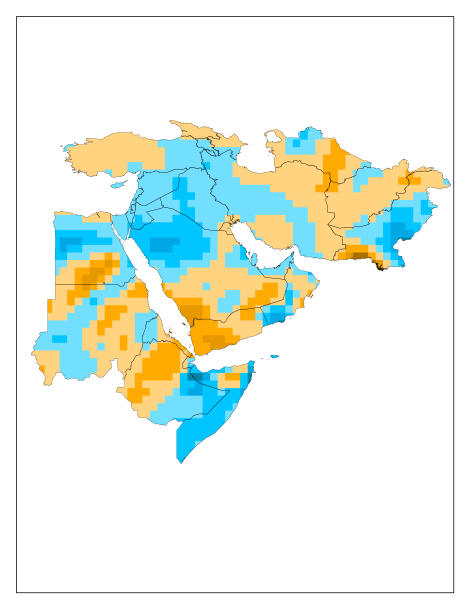 | 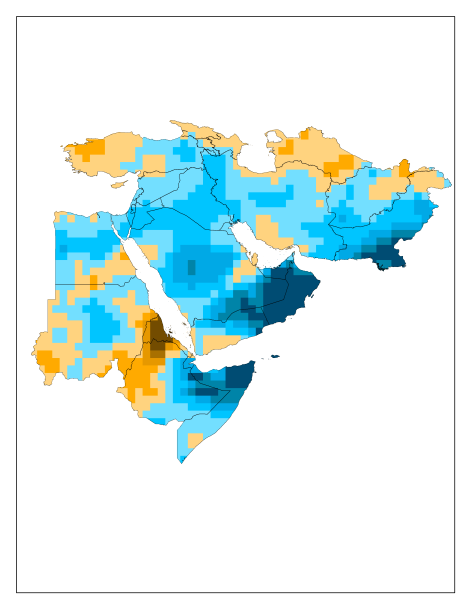 |
| 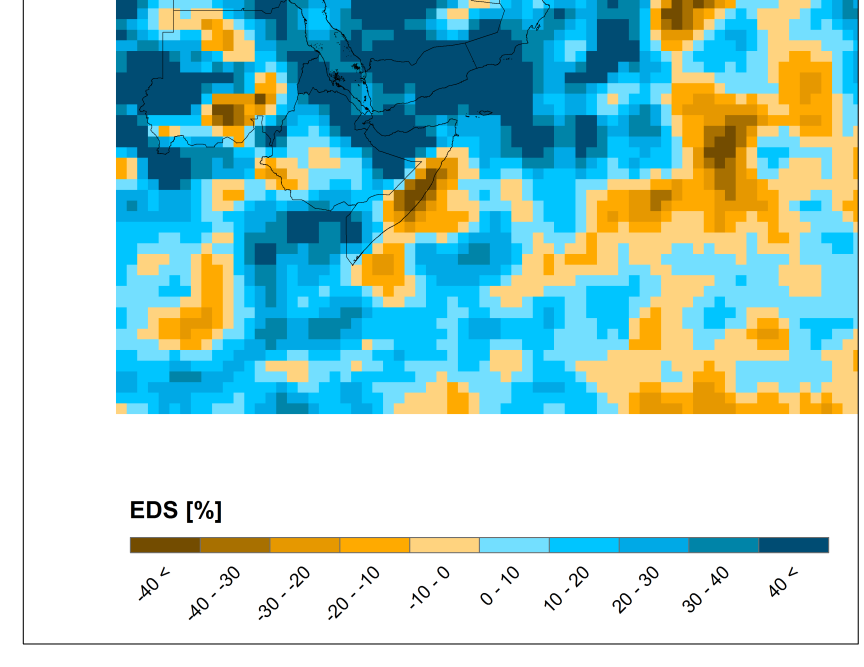 | | |

**Figure S8 | Ensemble downscaling signal of extreme and mean precipitation for RCP4.5 and RCP8.5 over the Middle East region for the spring season.** T=15yr, T=5yr and T=1yr refer to extreme precipitation of 15-, 5- and 1-year return periods, respectively. ‘Mean’ corresponds to mean precipitation. The maps were generated using the software ArcGIS (version 10) <http://www.esri.com/products>.

|  | **RCP4.5** | **RCP8.5** |
| --- | --- | --- |
| **15-yr** | 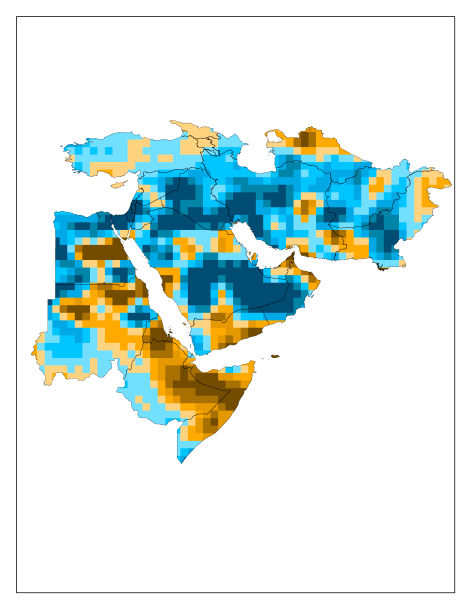 | 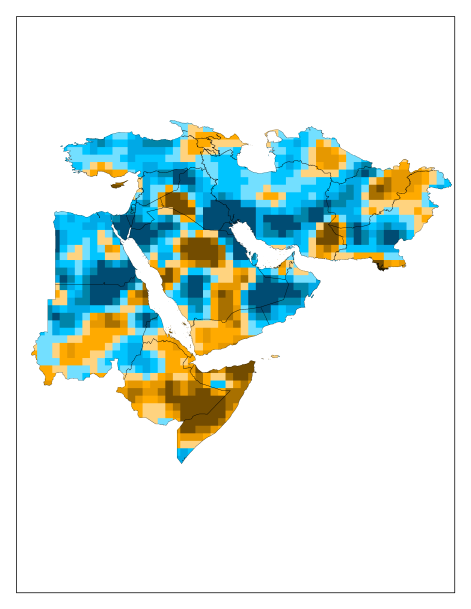 |
| **5-yr** | 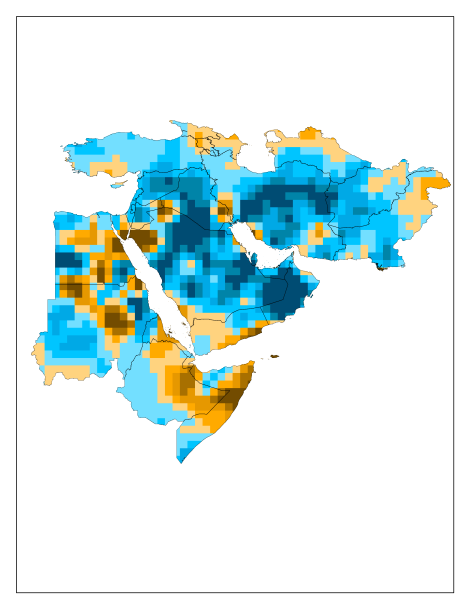 | 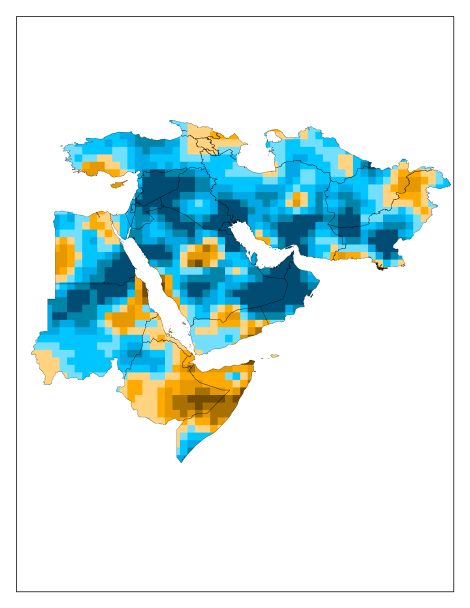 |
| **1-yr** | 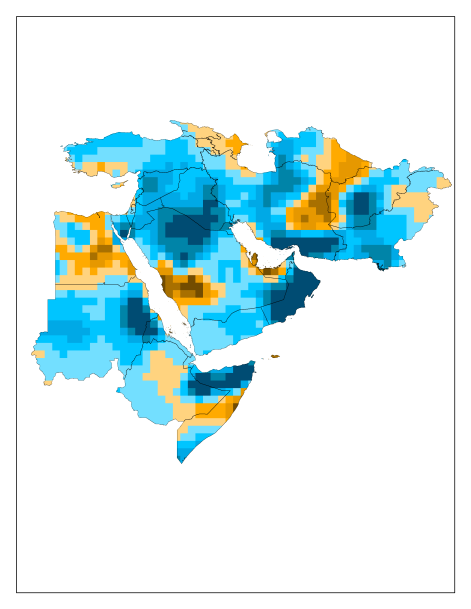 | 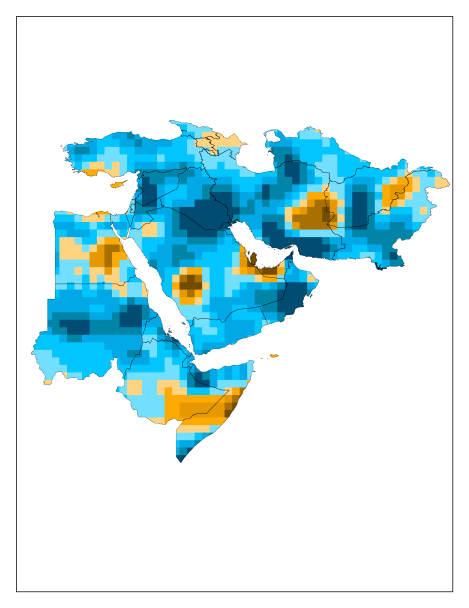 |
| **Mean** | 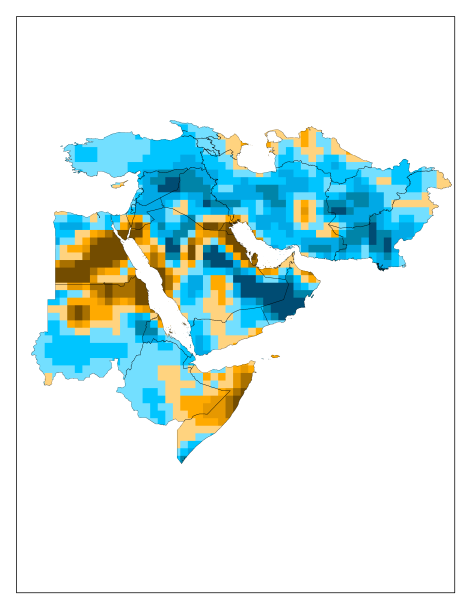 | 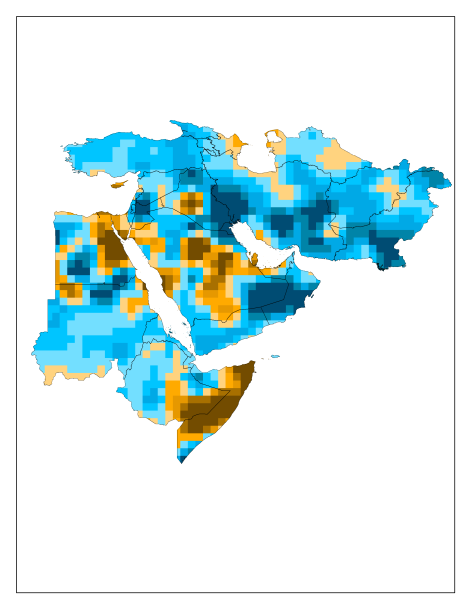 |
| 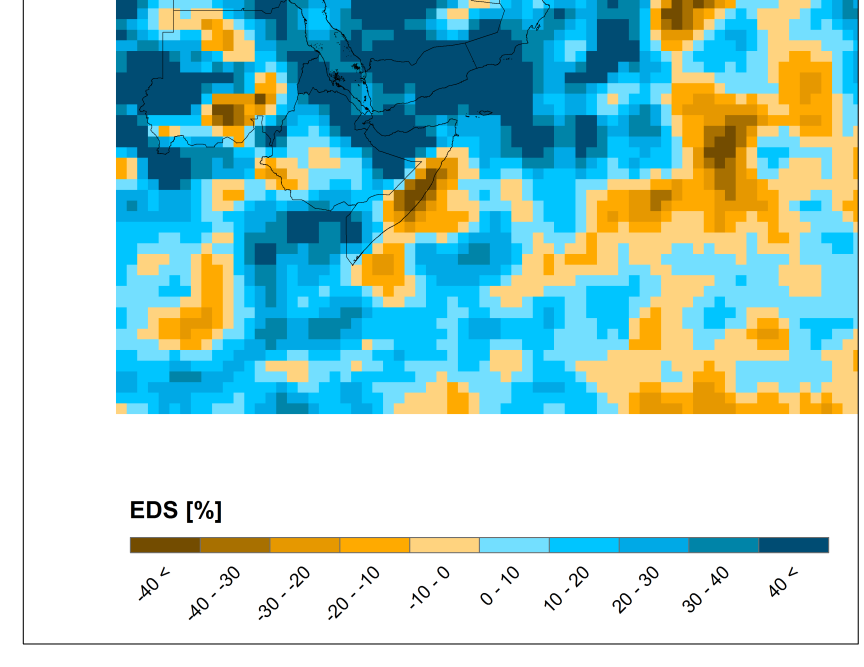 | | |

**Figure S9 | Ensemble downscaling signal of extreme and mean precipitation for RCP4.5 and RCP8.5 over the Middle East region for the summer season.** T=15yr, T=5yr and T=1yr refer to extreme precipitation of 15-, 5- and 1-year return periods, respectively. ‘Mean’ corresponds to mean precipitation. The maps were generated using the software ArcGIS (version 10) <http://www.esri.com/products>.

|  | **RCP4.5** | **RCP8.5** |
| --- | --- | --- |
| **15-yr** | 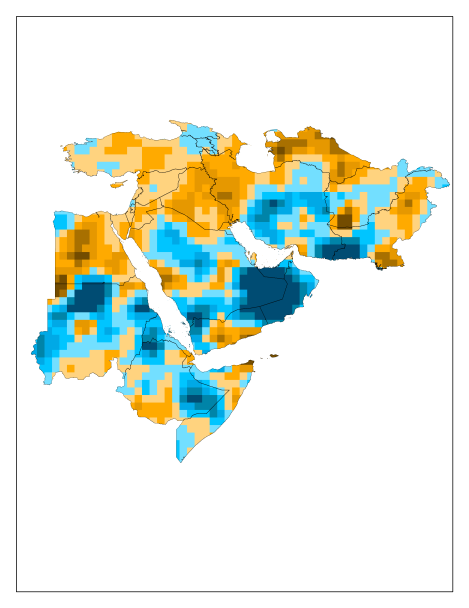 | 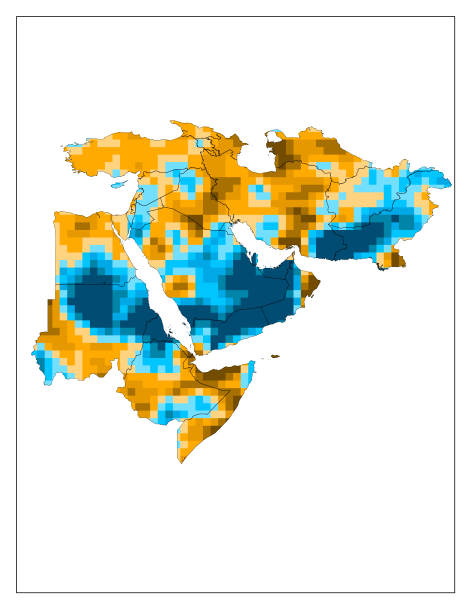 |
| **5-yr** | 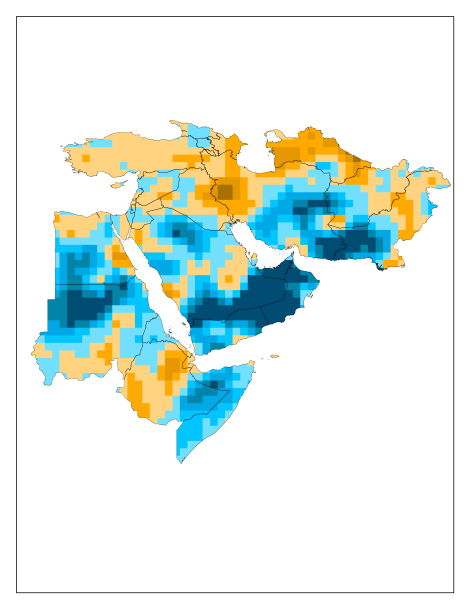 | 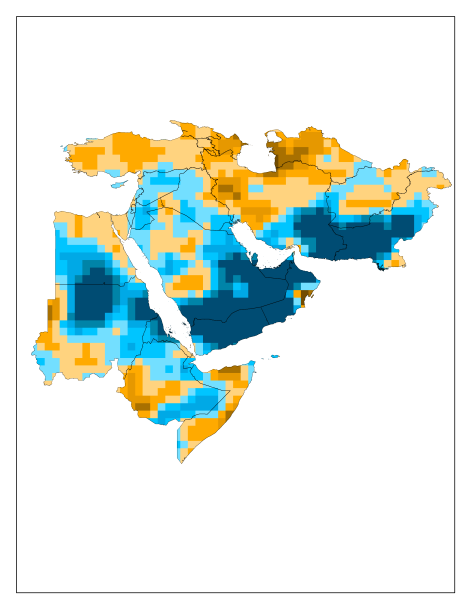 |
| **1-yr** | 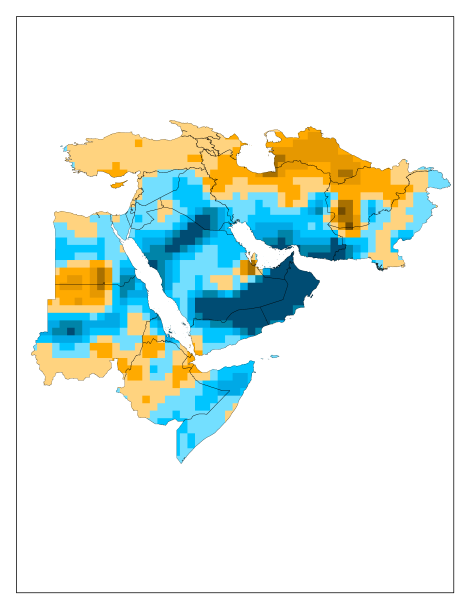 | 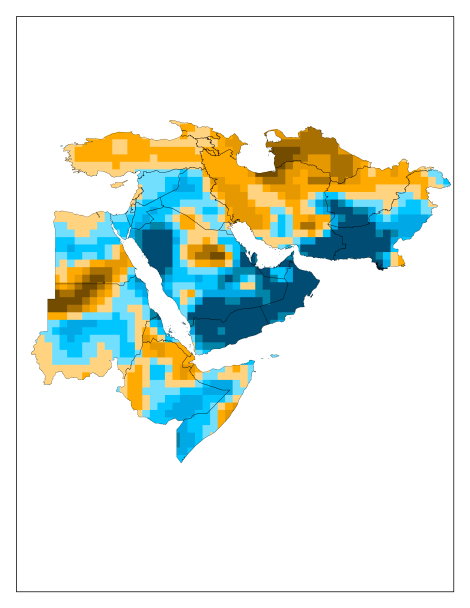 |
| **Mean** | 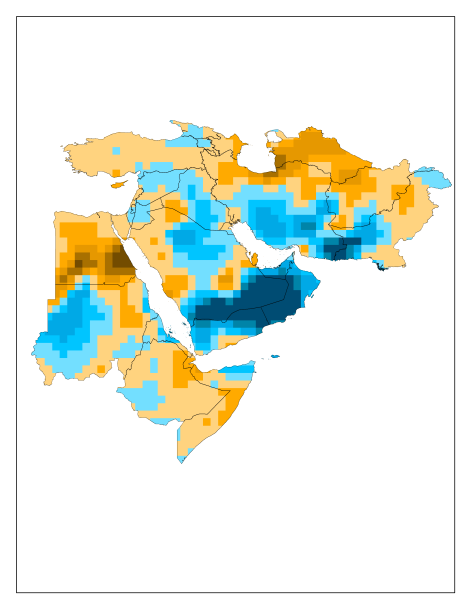 | 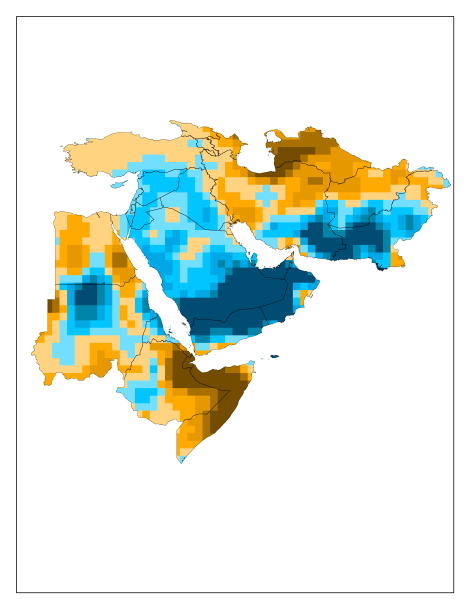 |
| 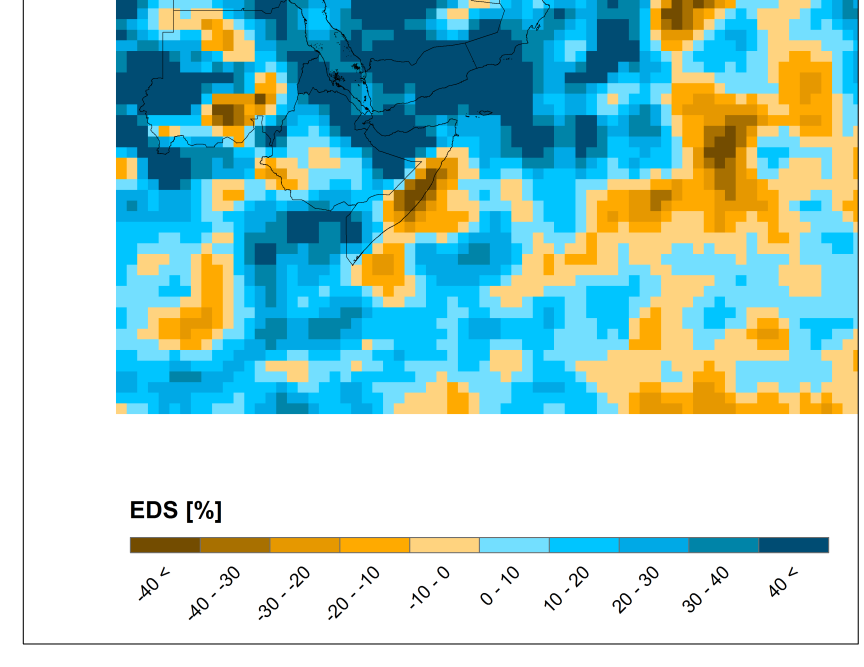 | | |

**Figure S10 | Ensemble downscaling signal of extreme and mean precipitation for RCP4.5 and RCP8.5 over the Middle East region for the autumn season.** T=15yr, T=5yr and T=1yr refer to extreme precipitation of 15-, 5- and 1-year return periods, respectively. ‘Mean’ corresponds to mean precipitation. The maps were generated using the software ArcGIS (version 10) <http://www.esri.com/products>.

**
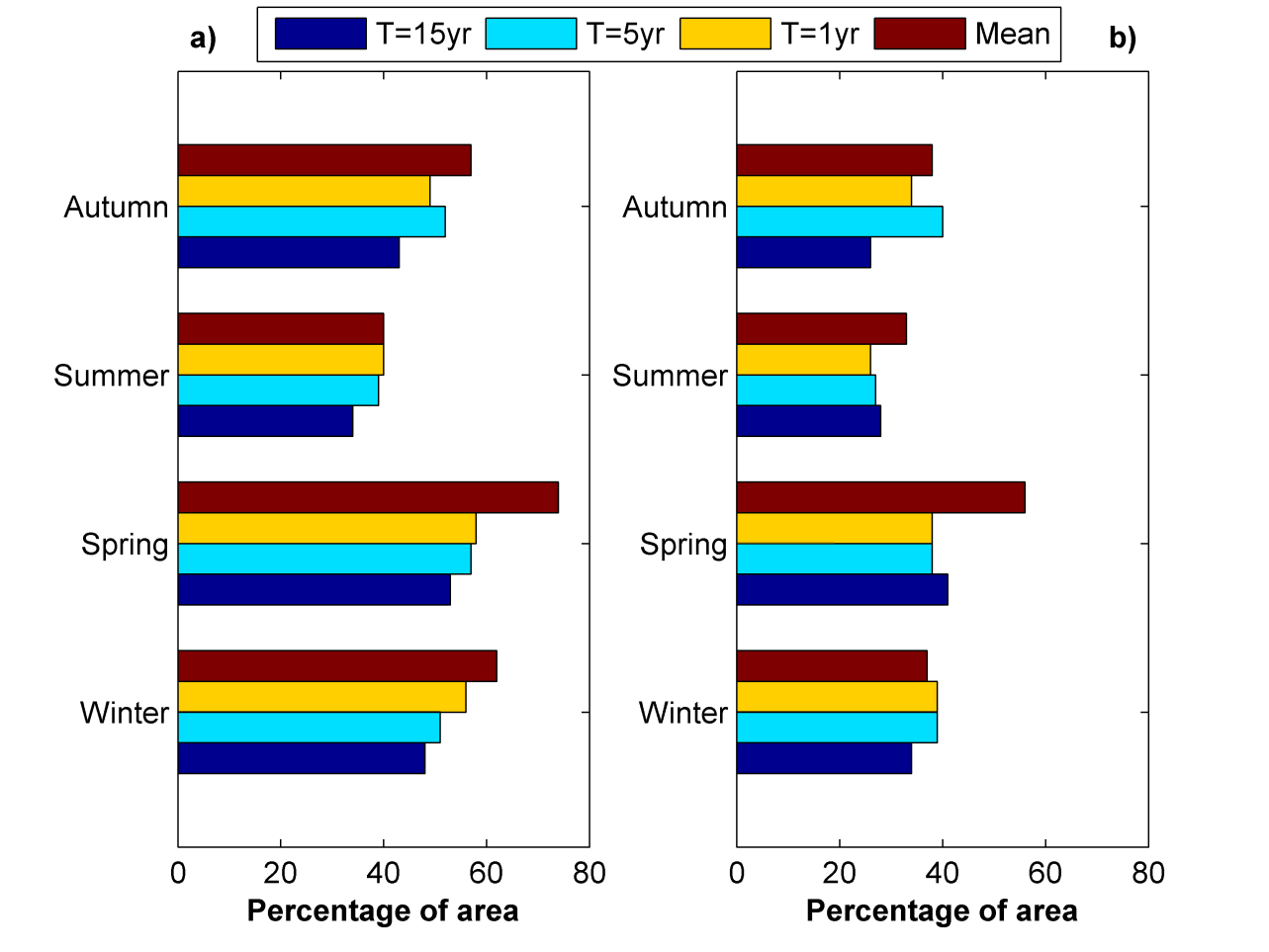
**

**Figure S11 | Percentage of the Middle East area with high consistency (-10 ≤ EDS ≤ 10) between the RCM and GCM results for mean and extreme precipitation for RCP4.5 (a) and RCP8.5 (b).** T=15yr, T=5yr and T=1yr refer to extreme precipitation of 15-, 5- and 1-year return periods, respectively. ‘Mean’ corresponds to mean precipitation. The plots were created using the software MATLAB (version 2013a) <http://www.mathworks.com/products/matlab/>.


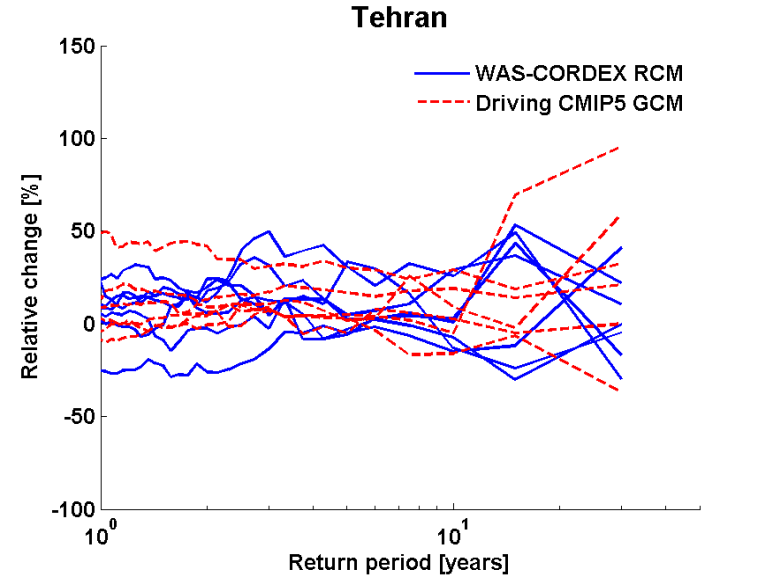

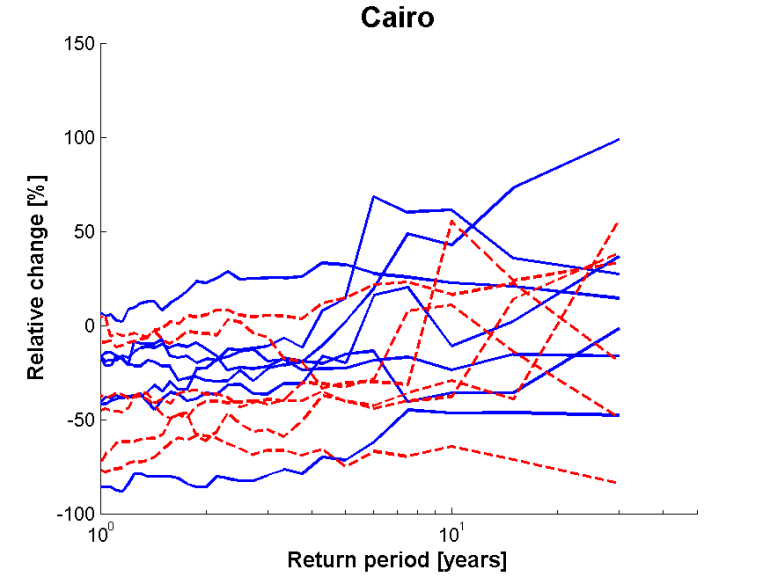


**Figure S12 | Comparison of spring extreme precipitation changes obtained from the WAS-CORDEX RCMs and the driving CMIP5 GCMs for some selected Middle Eastern capitals, for RCP8.5.** The plots were created using the software MATLAB (version 2013a) <http://www.mathworks.com/products/matlab/>.

**Table S1** List of the used WAS-CORDEX RCMs and driving CMIP5 GCMs and their attributes.

| WAS-CORDEX RCM | | | | Driving CMIP5 GCM | | | |
| --- | --- | --- | --- | --- | --- | --- | --- |
| Model name | Resolution | Institution | Reference | Model name | Resolution  (lon $\times$ lat) | Institution | Reference |
| REMO2009 | 0.44° $\times$ 0.44° | Climate Service Center (CSC),  Germany | Jacob  et al.^2^ | MPI-ESM-LR | 1.90° $\times$ 1.90° | Max Planck Institute  for Meteorology (MPI-M), Germany | Zanchettin  et al.^5^ |
| RCA4 | 0.44° $\times$ 0.44° | Swedish Meteorological  and Hydrological  Institute (SMHI),  Sweden | Samuelsson  et al.^3^  &  Kupiainen  et al.^4^ | CNRM-CM5 | 1.4° $\times$ 1.4° | Centre National  de Recherches Météorologiques, France | Voldoire  et al.^6^ |
| RCA4 |  |  |  | EC-EARTH | 1.12° $\times$ 1.12° | European EC-Earth  consortium | Hazeleger  et al.^7^ |
| RCA4 |  |  |  | IPSL-CM5A-MR | 2.5° $\times$ 1.25° | Institut Pierre-Simon  Laplace, France | Dufresne  et al.^8^ |
| RCA4 |  |  |  | MIROC5 | 1.4° $\times$ 1.4° | Atmosphere and Ocean  Research Institute, University of Tokyo, Japan | Watanabe  et al.^9^ |
| RCA4 |  |  |  | MPI-ESM-LR | 1.9° $\times$ 1.9° | Max Planck Institute  for Meteorology (MPI-M), Germany | Zanchettin  et al.^5^ |
| RCA4 |  |  |  | GFDL-ESM2M | 2.5° $\times$ 2° | Geophysical Fluid  Dynamics Laboratory, USA | Donner  et al.^10^ |

**References**

1. Hosseinzadehtalaei, P., Tabari, H. & Willems, P. Uncertainty assessment for climate change impact on intense precipitation: how many model runs do we need?. *Int. J. Climatol.* doi:10.1002/joc.5069 (2017).
2. Jacob, D. *et al.* Assessing the transferability of the regional climate model remo to different coordinated regional climate downscaling experiment (cordex) regions. *Atmosphere* **3**, 181–199 (2012).
3. Samuelsson, P. *et al.* The Rossby Centre regional climatemodel RCA3: model description and performance. *Tellus* **63A**, 4–23, (2011).
4. Kupiainen, M. *et al.* *Rossby Centre regional atmospheric model, RCA4*. Rossby Centre Newsletter, June (2011).
5. Zanchettin, D., Rubino, A., Matei, D., Bothe, O. & Jungclaus, J. H. Multidecadal-to-centennial SST variability in the MPIESM simulation ensemble for the last millennium. *Clim. Dynam.* **39**, 419–444 (2012).
6. Voldoire, A. *et al.* The CNRM-CM5.1 global climatemodel: description and basic evaluation. *Clim. Dynam.* **40**, 2091–2121 (2013).
7. Hazeleger W. *et al.* EC-Earth: a seamless Earth-system prediction approach in action. *Bull. Am. Meteorol. Soc.* **91**, 1357–1363 (2010).
8. Dufresne, J.-L., *et al.* Climate change projections using the IPSL-CM5 Earth system model: From CMIP3 to CMIP5. *Clim. Dyn.* **40**, 2123–2165 (2013).
9. Watanabe, M., *et al.* Improved climate simulation by MIROC5: Mean states, variability, and climate sensitivity. *J. Clim.* **23**, 6312–6335 (2010).
10. Donner, L. J. *et al.* The dynamical core, physical parameterizations, and basic simulation characteristics of the atmospheric component AM3 of the GFDL global coupled model CM3. *J. Clim.* **24**, 3484–3519 (2011).
